# Efficacy of Synaptic Inhibition Depends on Multiple, Dynamically Interacting Mechanisms Implicated in Chloride Homeostasis

**DOI:** 10.1371/journal.pcbi.1002149

**Published:** 2011-09-08

**Authors:** Nicolas Doyon, Steven A. Prescott, Annie Castonguay, Antoine G. Godin, Helmut Kröger, Yves De Koninck

**Affiliations:** 1 Division of Cellular and Molecular Neuroscience, Centre de recherche Université Laval Robert-Giffard, Québec, Québec, Canada; 2 Department of Psychiatry & Neuroscience, Université Laval, Québec, Québec, Canada; 3 Department of Neurobiology and Pittsburgh Center for Pain Research, University of Pittsburgh, Pittsburgh, Pennsylvania, United States of America; 4 Department of Physics, Université Laval, Québec, Québec, Canada; University of Freiburg, Germany

## Abstract

Chloride homeostasis is a critical determinant of the strength and robustness of inhibition mediated by GABA_A_ receptors (GABA_A_Rs). The impact of changes in steady state Cl^−^ gradient is relatively straightforward to understand, but how dynamic interplay between Cl^−^ influx, diffusion, extrusion and interaction with other ion species affects synaptic signaling remains uncertain. Here we used electrodiffusion modeling to investigate the nonlinear interactions between these processes. Results demonstrate that diffusion is crucial for redistributing intracellular Cl^−^ load on a fast time scale, whereas Cl^−^extrusion controls steady state levels. Interaction between diffusion and extrusion can result in a somato-dendritic Cl^−^ gradient even when KCC2 is distributed uniformly across the cell. Reducing KCC2 activity led to decreased efficacy of GABA_A_R-mediated inhibition, but increasing GABA_A_R input failed to fully compensate for this form of disinhibition because of activity-dependent accumulation of Cl^−^. Furthermore, if spiking persisted despite the presence of GABA_A_R input, Cl^−^ accumulation became accelerated because of the large Cl^−^ driving force that occurs during spikes. The resulting positive feedback loop caused catastrophic failure of inhibition. Simulations also revealed other feedback loops, such as competition between Cl^−^ and pH regulation. Several model predictions were tested and confirmed by [Cl^−^]_i_ imaging experiments. Our study has thus uncovered how Cl^−^ regulation depends on a multiplicity of dynamically interacting mechanisms. Furthermore, the model revealed that enhancing KCC2 activity beyond normal levels did not negatively impact firing frequency or cause overt extracellular K^−^ accumulation, demonstrating that enhancing KCC2 activity is a valid strategy for therapeutic intervention.

## Author Summary

Fast synaptic inhibition relies on chloride current to hyperpolarize the neuron or to prevent depolarization caused by concurrent excitatory input. Both scenarios necessarily involve chloride flux into the cell and, thus, a change in intracellular chloride concentration. The vast majority of models neglect changes in ion concentration despite experimental evidence that such changes occur and are not inconsequential. The importance of considering chloride homeostasis mechanisms is heightened by evidence that several neurological diseases are associated with deficient chloride extrusion capacity. Steady state chloride levels are altered in those disease states. Fast chloride dynamics are also likely affected, but those changes have yet to be explored. To this end, we built an electrodiffusion model that tracks changes in the concentration of chloride plus multiple other ion species. Simulations in this model revealed a multitude of complex, nonlinear interactions that have important consequences for the efficacy of synaptic inhibition. Several predictions from the model were tested and confirmed with chloride imaging experiments.

## Introduction

In the central nervous system, fast inhibition is mediated by GABA_A_ and glycine receptor-gated Cl^−^ channels (GABA_A_R and GlyR). Influx of Cl^−^ through these channels produces outward currents that cause hyperpolarization or prevent depolarization caused by concurrent excitatory input (*i.e.* shunting) [1], [2]. Hyperpolarization and shunting both typically reduce neuronal spiking. However, Cl^−^ influx through GABA_A_R necessarily increases [Cl^−^]_i_, which in turn causes depolarizing shifts in the Cl^−^ reversal potential (*E*_Cl_) [3], [4]. As the Cl^−^ gradient is depleted and *E*_Cl_ rises, the efficacy of GABA_A_R-mediated control of spiking is compromised [5]. Therefore, mechanisms that restore the transmembrane Cl^−^ gradient are crucial for maintaining the efficacy of GABA_A_R-mediated inhibition.

Cation-chloride cotransporters (CCCs) play a key role in maintaining the Cl^−^ gradient across the membrane [6], [7]. Most relevant to neurons are the Na^+^-K^+^-2Cl^−^ cotransporter (NKCC1), which normally mediates uptake of Cl^−^ [8], and the K^+^-Cl^−^ cotransporter, isoform 2, (KCC2), which normally extrudes Cl^−^. Interestingly, a reduction in KCC2 expression and/or function is involved in the pathogenesis of several neurological disorders, including epilepsy and neuropathic pain [9]–[15]. Motivated by the clinical relevance of hyperexcitability caused by changes in KCC2 activity, conductance-based compartmental models have been used to study how changes in *E*_Cl_ influence inhibitory control of neuronal spiking [5].

*E*_Cl_ can change as a result of altered KCC2 expression or activity [7], [16], [17]. *E*_Cl_ can also change dynamically, on a fast time scale, as a result of Cl^−^ flux through GABA_A_ receptors, particularly in small structures like distal dendrites [2], . If *E*_Cl_ changed only slowly, it could be reasonably approximated as static relative to other neuronal processes occurring on a faster time scale; however, since *E*_Cl_ changes rapidly, it may interact in potentially complex ways with important neuronal processes like synaptic integration. To investigate those interactions, one must treat [Cl^−^] as a dynamical quantity evolving in space and time.

**Figure 1:**
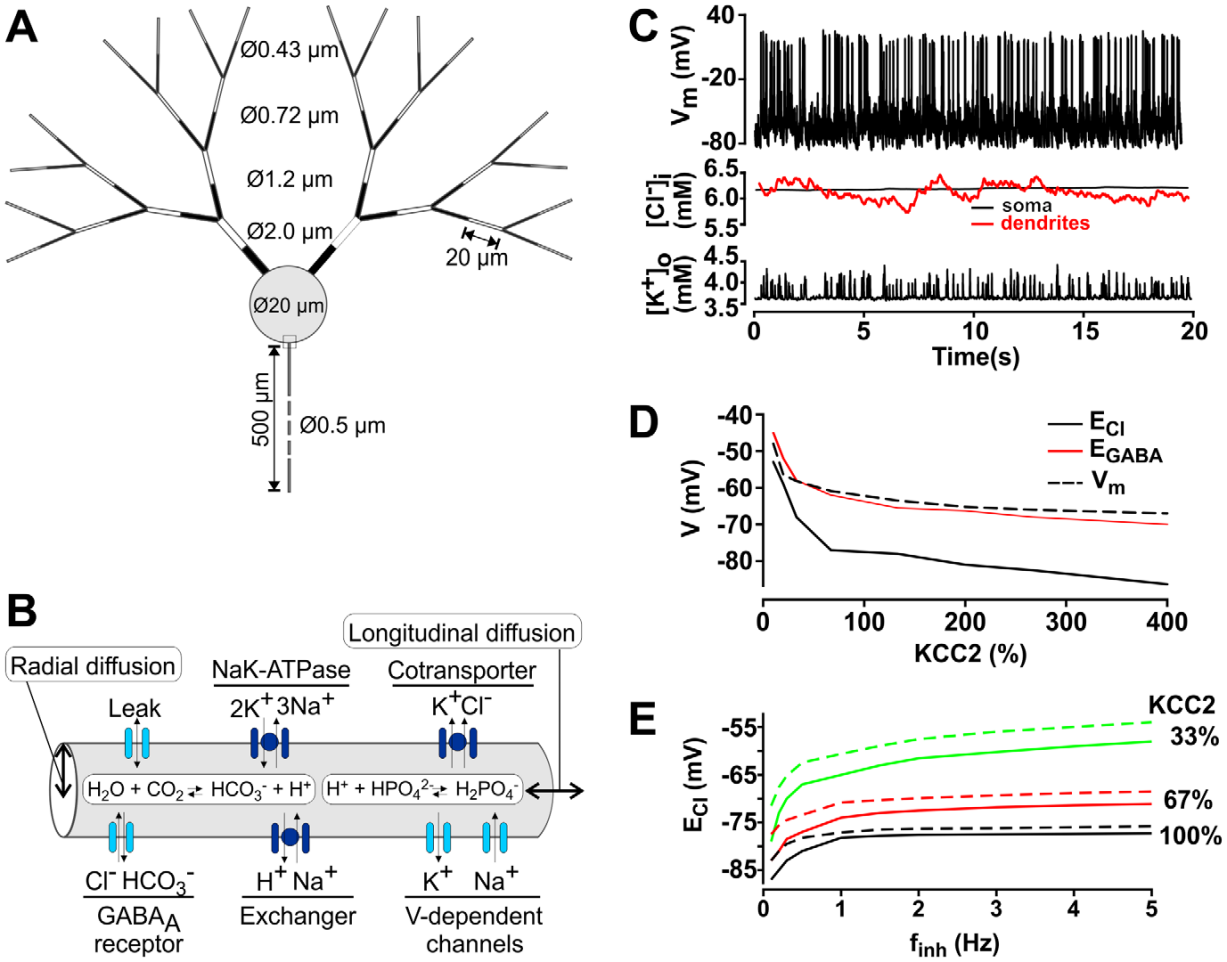
Summary and initial validation of model. **A**. Schematic of model neuron showing geometry and compartment dimensions. **B.** Summary of ion flux mechanisms included in the model (see Methods for details). Diffusion in the extracellular space is not depicted. **C.** Sample traces of membrane potential together with [K^+^]_o_ measured in the extracellular shell surrounding the soma and [Cl^−^]_i_ measured in the soma (black) and in a dendrite (red). This is only a subset of ion species whose concentrations were continuously monitored in all compartments, and from which reversal potentials were continuously updated. **D.** As predicted, reducing KCC2 below its “normal” level (100%) caused large depolarizing shifts in *E*_Cl_ and *E*_GABA_, whereas increasing KCC2 up to 400% above normal caused only minor hyperpolarizing shifts. Simulation includes background synaptic input with *f*_inh_  =  0.8 Hz and *f*_exc_  =  0.2 Hz/synapse. The dashed line represents the mean value of membrane potential averaged over 200 s. **E.** Reversal potentials also depended on the rate of GABA_A_R input, which dictates the Cl^−^ load experienced by the neuron. Increasing *f*_inh_ caused a depolarizing shift in *E*_Cl_, the extent of which increased when KCC2 was decreased. For these simulations, *f*_inh_/*f*_exc_ ratio was fixed at 4 and *f*_inh_ was varied from 0.05 Hz to 4.8 Hz. Dashed lines represent results from simulations performed with tonic GABA_A_ conductances while solid lines represent simulations performed without it.

The spatio-temporal dynamics of [Cl^−^]_i_ depend on several factors, including GABA_A_R-mediated Cl^−^ flux, longitudinal diffusion within dendrites and the soma, and CCC activity. Furthermore, Cl^−^ dynamics involve complex non-linear interactions with other ion species, which have been overlooked by previous models [19]. To understand how these dynamical processes interact with each other, we built an electrodiffusion model that monitors intra- and extracellular concentrations of several ion species (Cl^−^, Na^+^, K^+^, Ca^2+^, HCO_3_^−^, H^+^, HPO_4_^2−^, H_2_PO_4_^−^) across neuronal compartments (see Fig. 1A–C). Our model revealed several consequences of impaired Cl^−^ extrusion on neuronal function, including a positive feedback loop between intracellular Cl^−^ accumulation and excitatory activity or spiking that can lead to catastrophic failure of inhibition. Several predictions of the model were confirmed by direct measurement of [Cl^−^]_i_, by fluorescence lifetime imaging microscopy (FLIM).

## Results

### *E*_Cl_ and *E*_GABA_ depend non-linearly on KCC2 activity and synaptic input

Past experiments have established that Cl^−^ extrusion via KCC2 plays a crucial role in maintaining the values of *E*_Cl_ and *E*_GABA_ below the resting membrane potential [20], but they have not established how KCC2 activity relates quantitatively to *E*_Cl_ and *E*_GABA_, in particular under conditions of ongoing, distributed synaptic input. Therefore, as a first step, we varied KCC2 activity and measured the impact on *E*_Cl_ and *E*_GABA_ (measured at the soma) in a model neuron receiving a fixed level of background excitatory and inhibitory synaptic input (Fig. 1D). Values of *E*_Cl_ and *E*_GABA_ in middle and distal dendrites are described by similar curves shifted to slightly more depolarized values (data not shown) consistent with the somato-dendritic gradient described below. This is important since neurons *in vivo* are bombarded by synaptic activity [21], but it remains unclear how this may affect *E*_Cl_ and, in turn, be affected by *E*_Cl_. Consistent with qualitative experimental findings [9], [22], [23], both reversal potentials underwent depolarizing shifts as KCC2 activity was reduced, with *E*_Cl_ approaching the mean membrane potential (Fig. 1D). Notably, *E*_GABA_ was less negative than *E*_Cl,_, especially at high values of KCC2 activity, consistent with *E*_GABA_ depending jointly on [Cl^−^]_i_ and [HCO_3_^−^]_i_ [24]. However, unlike the large depolarizing shift in *E*_Cl_ caused by reducing KCC2 activity, increasing KCC2 activity beyond its normal value caused only a marginal hyperpolarizing shift in *E*_Cl_, which approached the K^+^ reversal potential (*E*_K_) near -90 mV. This is consistent with KCC2 normally operating near equilibrium. Hence, while reduction in KCC2 activity can cause strong reduction of inhibition, excess KCC2 activity has a limited influence on the strength of inhibition, insofar as we assume that strength of GABA_A_R-mediated inhibition is a function of the value of *E*_GABA_.

Thus, in addition to validating our model, this first set of simulations revealed an interesting nonlinear relationship between KCC2 activity and *E*_Cl_. However, we expected that *E*_Cl_ should depend not only on KCC2, but also on factors like GABA_A_R input – this was the main motivation for developing an electrodiffusion model. As a preliminary test, we varied the rate of inhibitory synaptic input together with KCC2 activity. Results show that *E*_Cl_ underwent a depolarizing shift, the magnitude of which depended on KCC2 activity, as the rate of inhibitory input increased (Fig. 1E). At a normal KCC2 level, increasing the activation rate of GABA_A_R synapses from 0.2 to 5 Hz drove *E*_Cl_ up by only 7 mV, whereas the same change in activation rate drove *E*_Cl_ up by 24 mV when KCC2 activity was decreased to 33% of its normal value. Thus, KCC2 activity not only controls baseline *E*_Cl_, it also determines how stably *E*_Cl_ is maintained when the Cl^−^ load is increased by synaptic input. Tonic inhibition due to activation of extrasynaptic GABA_A_ receptors by ambient GABA can also contribute to intracellular Cl^−^ accumulation and depolarize *E*_Cl_. To test the impact of tonic inhibition, we performed simulations with and without this form of inhibition. Results obtained with and without tonic inhibition were qualitatively the same (Fig. 1E).

**Figure 2:**
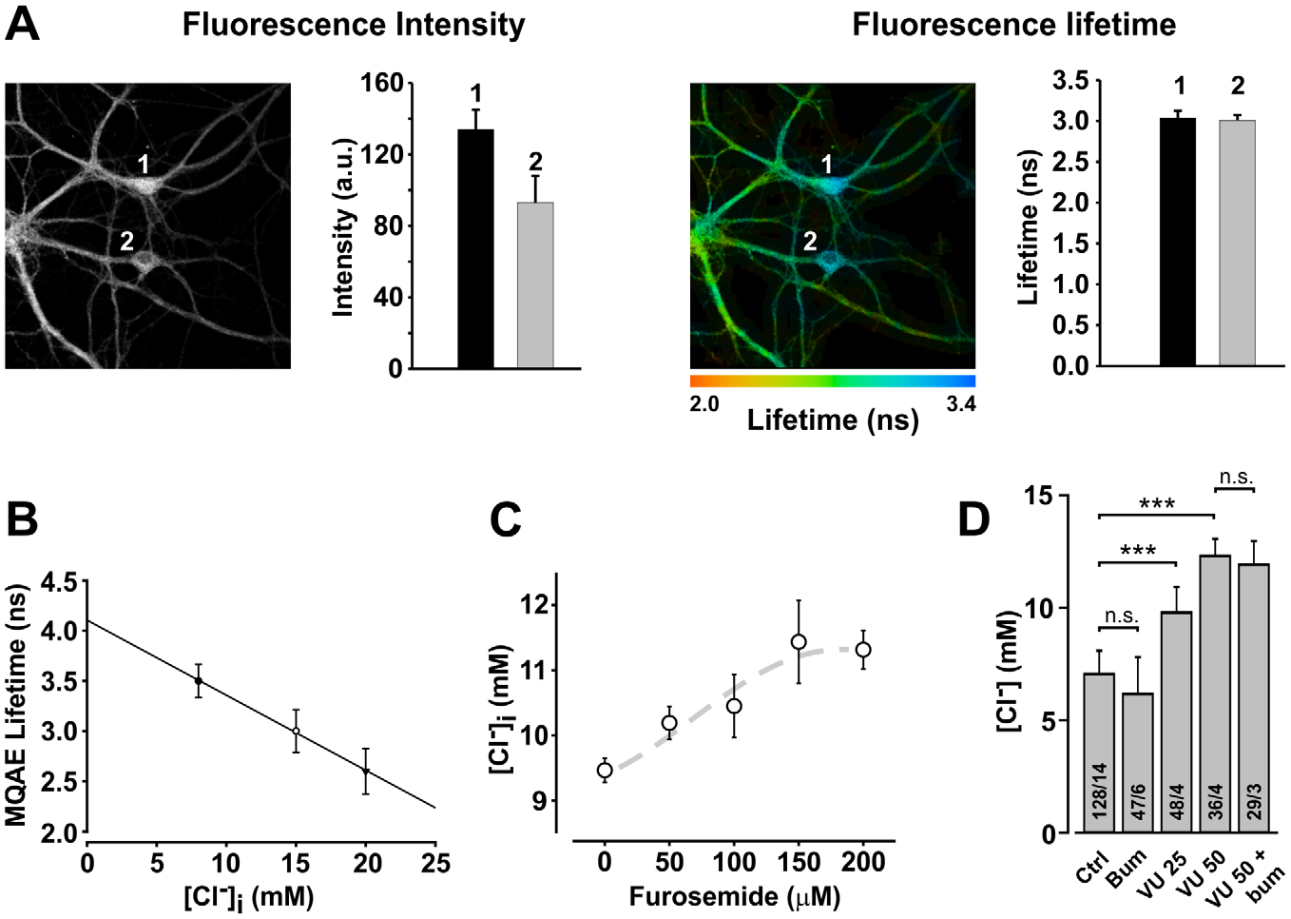
Measurements of [Cl^−^]_i_ in neurons with MQAE using Fluorescence Lifetime Imaging Microscopy (FLIM). **A.** Two-photon excitation fluorescence of MQAE-loaded hippocampal neurons (26 DIV). The mean intensity of MQAE fluorescence within the cell bodies 1 & 2 was significantly different (*left*), which could be interpreted as indicating different levels of [Cl^−^]_i_ or different dye uptake and accumulation between the two cells. The lifetime maps of the same cells are shown in the micrograph on the *right*. Note how, in contrast to intensities, the fluorescence lifetime of both cells were not significantly different indicating that there were no difference in [Cl^−^]_i_ between the two cells. Values are mean ± S.D. of all pixels in each cell body. **B.** Measurements of MQAE lifetime at different [Cl^−^]_i_ inside the cell body after membrane permeabilization and equilibration with [Cl^−^]_o_ at 8, 15 or 20 mM (N  =  73 cells/12 coverslips). According to the Stern-Volmer equation: τ_0_/τ  =  1 + Ksv[Cl^−^]. The measured Ksv from these data was 32 M^−1^, consistent with previous reports [86]. **C**. Effect of increasing concentration of furosemide (to block KCC2) on [Cl^−^]_i_ in cultured neurons exposed to 100 µM muscimol (to evoke a constant Cl^−^ load by opening GABA_A_R; N  =  75 cells/10 coverslips). **D**. The selective KCC2 antagonist VU 0240551 caused a dose-dependent significant increase in [Cl^−^]_i_ (p<0.05), but bumetamide had no significant (n.s.) effect alone or after blocking KCC2 with VU 0240551, indicating lack of significant NKCC1 transport in these cells (N indicated in each bar  =  cells/coverslips; ***, p < 0.001).

To test experimentally the impact of the level of KCC2 activity on intracellular Cl^−^ accumulation, we loaded neurons in primary cultures (>21 days in vitro; DIV) with MQAE and measured changes in [Cl^−^]_i_ using FLIM. FLIM measurements have the advantage of being unbiased by the amount of indicator from cell to cell (Fig. 2A, B), minimizing the variability between measurements as well as shielding the measurements from changes in cell volumes [25]. We first bath applied the GABA_A_R agonist muscimol to trigger Cl^−^ influx through GABA_A_R channels. We then applied various concentrations of furosemide or VU 0240551 for 20 minutes to block KCC2 activity. In the presence of Cl^−^ load through activated GABA_A_ channels, application of furosemide or VU 0240551 led to dose-dependent Cl^−^ accumulation (Fig. 2C, D), in agreement with the predictions of simulations (cf. Fig. 1D). At high doses, furosemide can antagonize both KCC2 and NKCC1; however, at > 21 DIV, hippocampal neurons in culture are generally thought to fully express KCC2 but to no longer express NKCC1 [6]. To test this, we used bumetanide at a concentration (50 µM) where it selectively blocks NKCC1. Administration of bumetanide to cells exposed to muscimol cause no change in [Cl^−^]_i_ (Fig. 2D). The presence of significant Cl^−^ export through KCC2 may however mask any NKCC1-mediated Cl^−^ import. To test for this, we blocked KCC2 with the recently developed selective blocker VU 0240551 [25]. Further addition of bumetanide after KCC2 blockade had no effect on [Cl^−^]_i_, confirming absence of significant NKCC1-mediated transport in these neurons (Fig. 2D). These results indicate **a)** significant KCC2 co-transport in > 21 DIV hippocampal neurons in culture, maintaining [Cl^−^]_i_ at a low level, and **b)** that both furosemide and VU 0240551 could be used under these conditions to selectively block KCC2-mediated transport.

With the importance of nonlinear interaction between GABA_A_R activity and KCC2 activity for intracellular Cl^−^ regulation thus established, we moved onto more detailed analysis of how Cl^−^ flux impacts the efficacy of synaptic inhibition.

### Transmembrane Cl^−^ gradient may vary between cellular compartments depending on the spatial distribution of synaptic input and cotransporter activity

**Figure 3:**
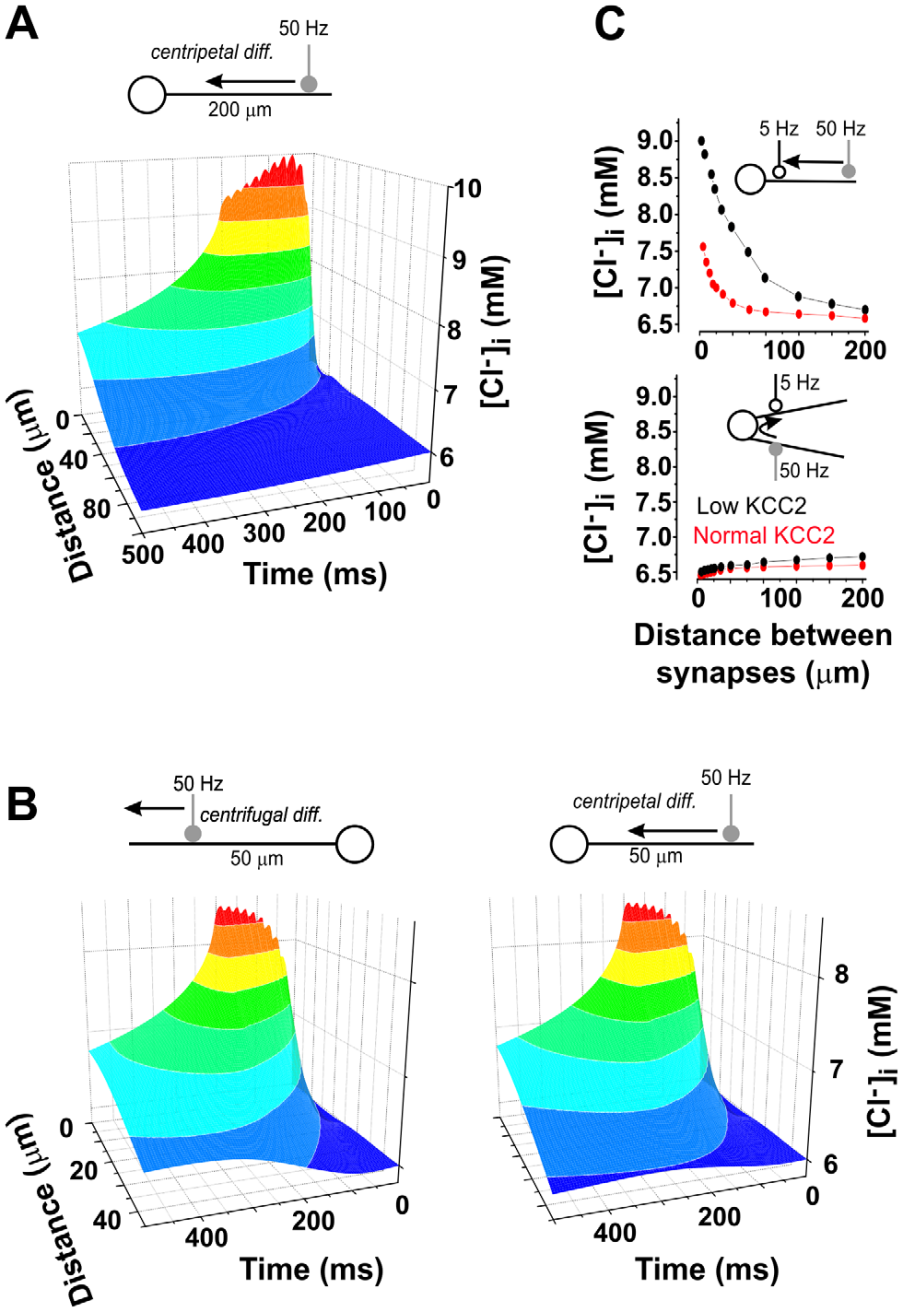
Redistribution of intracellular Cl^−^ through electrodiffusion. **A.** Chloride influx through a single GABA_A_R synapse (located at 200 µm from the soma) activated at 50 Hz produced a substantial longitudinal gradient in [Cl^−^]_i_ extending 60 µm on each side of the input. **B.** Chloride influx through a single GABA_A_R synapse (this time located at 50 µm from the soma) produced a longitudinal gradient which is steeper toward the soma (centripetal diffusion) than away from the soma (centrifugal diffusion). For this simulation, we used dendrites with constant diameters to ensure that the difference between left and right panels is due to the sink effect of the soma and not to the conical shape of the dendrite. We also lengthened the dendrite and increased the number of compartments to 60 compared to the cell geometry summarized in Fig. 1. **C.** Spread of Cl^−^ entering through one synapse (activated at 50 Hz) to a second “test” synapse (activated at 5 Hz) at varying inter-synapses distances was measured for normal and low (10%) KCC2 levels. Both synapses were activated simultaneously. For synapses positioned on the same primary dendrites (upper panel), the test synapse experienced a sizeable increase in [Cl^−^]_i_, especially when KCC2 was reduced, but there was no appreciable spread of Cl^−^ between synapses located on different primary dendrites (lower panel).

Spatial variation in *E*_Cl_ (or *E*_GABA_) between cellular compartments has been observed in several experiments [20], [26]–[29] but it is not typically accounted for in conventional neuron models. While a longitudinal, axo-somato-dendritic [Cl^−^]_i_ gradient could be due to differentially distributed cotransporter activity, it could also arise from intense focal GABA_A_R-mediated input. To test the latter scenario, we simulated high frequency GABA_A_R-mediated input to a single dendritic synapse and measured [Cl^−^]_i_ at different distances from the synapse at different times after the onset of input (Fig. 3A). Under the conditions tested, a GABA_A_R synapse activated at 50 Hz produced a longitudinal [Cl^−^]_i_ gradient of 50 µM/µm, which extended as far as 60 µm and could yield changes in *E*_GABA_ on the order of 5 mV within 200 ms (Fig. 3A). There were only subtle differences between centripetal and centrifugal diffusion (*i.e.* toward or away from soma, respectively; Fig. 3B). According to these data, if a GABA_A_ synapse receives sustained high frequency input, [Cl^−^]_i_ will increase near that synapse, influencing *E*_GABA_ at the original synapse as well at nearby synapses. This was further investigated by placing a “test” GABA_A_ synapse (activated at 5 Hz) at varying distances from the original GABA_A_ synapse (activated at 50 Hz). Both synapses were activated simultaneously. As predicted, *E*_GABA_ at the test synapse was affected by other GABA_A_R-mediated input on the same dendrite as far away as 50 µm (Fig. 3C *top*), or even farther when KCC2 activity was reduced. However, interactions also depended on synapse position relative to the neuron topology; for instance, synapses in relatively close proximity but located on different primary dendrites exhibited little if any interaction (Fig. 3C *bottom*), consistent with the soma acting as a sink that clamps [Cl^−^]_i_.

**Figure 4:**
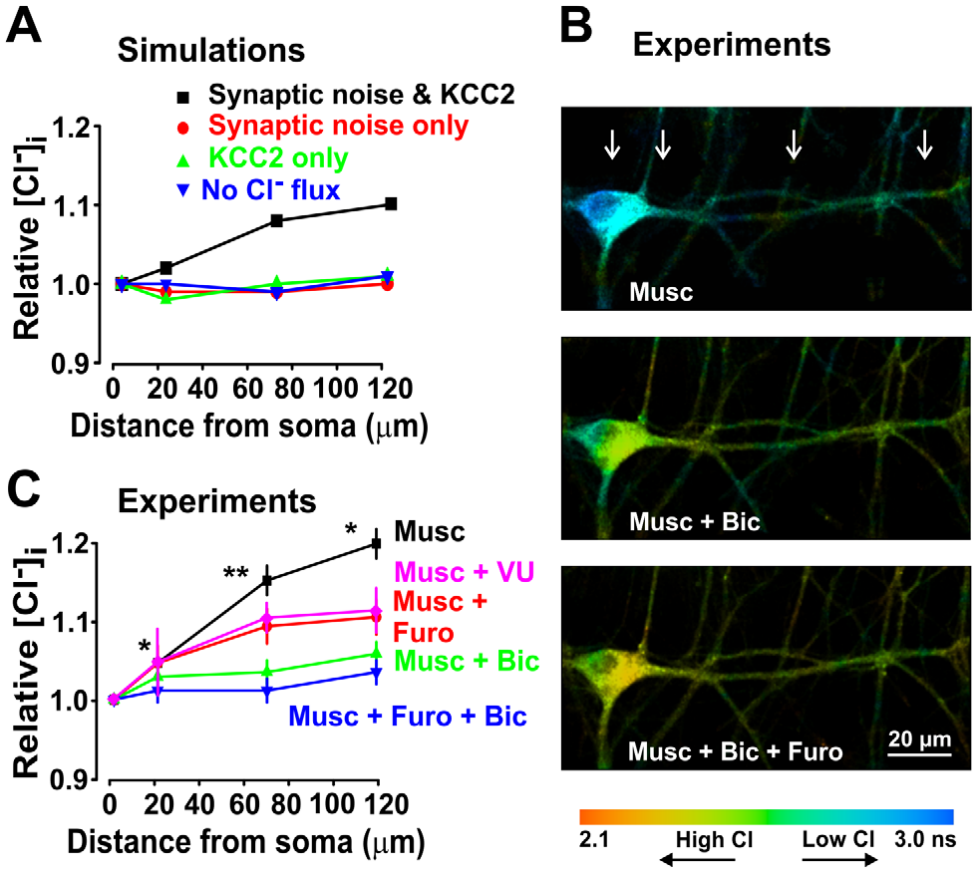
A standing somato-dendritic Cl^−^ gradient is caused by the joint action of KCC2 activity and GABA_A_R mediated synaptic input. **A.** Distribution of [Cl^−^]_i_ in a modeled dendrite as a function of distance from the soma in the presence and absence of Cl^−^ load due to distributed synaptic activity and of Cl^−^ extrusion through uniformly distributed KCC2. **B**. Photomicrographs of an example cell loaded with MQAE with lifetime color coding (blue: low Cl^−^ concentration, red: high Cl^−^ concentration). Intracellular Cl^−^ concentration was measured in the presence of muscimol (Musc; 100 µM) and/or bicuculline (Bic; 100 µM) and/or furosemide (Furo; 200 µM) and/or VU 0240551 (VU, 15 µM). Arrows indicate the location where measurements were performed. **C**. Effect of tonic activation of GABA_A_Rs by muscimol on [Cl^−^]_i_ in real dendrites as a function of distance from the soma (each data point represent mean ± SEM taken from 10–12 neurons; values from several dendrites were averaged for each cell). Bicuculline and/or furosemide and/or VU 0240551 were added to block Cl^−^ loading and extrusion, respectively.

Under *in vivo* conditions, neurons are known to be constantly bombarded by synaptic input [30]. We therefore tested whether this synaptic noise affects [Cl^−^]_i_ differently depending on the cellular compartment. We performed simulations in the presence or absence of KCC2 activity and in the presence or absence of synaptic noise. Simulations of distributed ongoing synaptic input with KCC2 distributed uniformly across the cell compartments yielded a clear somato-dendritic [Cl^−^]_i_ gradient (Fig. 4A *black*). In contrast, in the absence of simulated synaptic noise, there was no significant somato-dendritic [Cl^−^]_i_ gradient despite the presence of KCC2 (Fig. 4A *green*). Lack of a significant somato-dendritic [Cl^−^]_i_ gradient was also observed in the reverse scenario, i.e. in the presence of synaptic noise but without KCC2 (Fig. 4A *red*). Thus, a significant somato-dendritic [Cl^−^]_i_ gradient can exist when there is ongoing Cl^−^ influx, redistribution of that Cl^−^ load via diffusion, and Cl^−^ extrusion by KCC2. This clearly demonstrates that differential extrusion, i.e. inhomogeneous KCC2 density (see below), is not necessary for inhomogeneous transmembrane Cl^−^ gradients.

To test the predictions made by the model, we used FLIM to measure [Cl^−^]_i_ in MQAE-loaded neurons in culture (Fig. 4B). To mimic distributed Cl^−^ influx across the dendritic tree, we exposed the cultures to 100 µM muscimol. FLIM measurements indicated a significant [Cl^−^]_i_ gradient along dendrites (Fig. 4B *top*) which was either reduced by bicuculline (Fig. 4B *middle* and 4C) or blocked by the addition of furosemide or the recently developed more specific KCC2 inhibitor VU 0240551 [25] (Fig. 4B *bottom* and 4C), consistent with predictions from simulations (cf. Fig. 4A). The small remaining gradient in the presence of furosemide may indicate the presence of another chloride transport mechanism not accounted for in the model.

**Figure 5:**
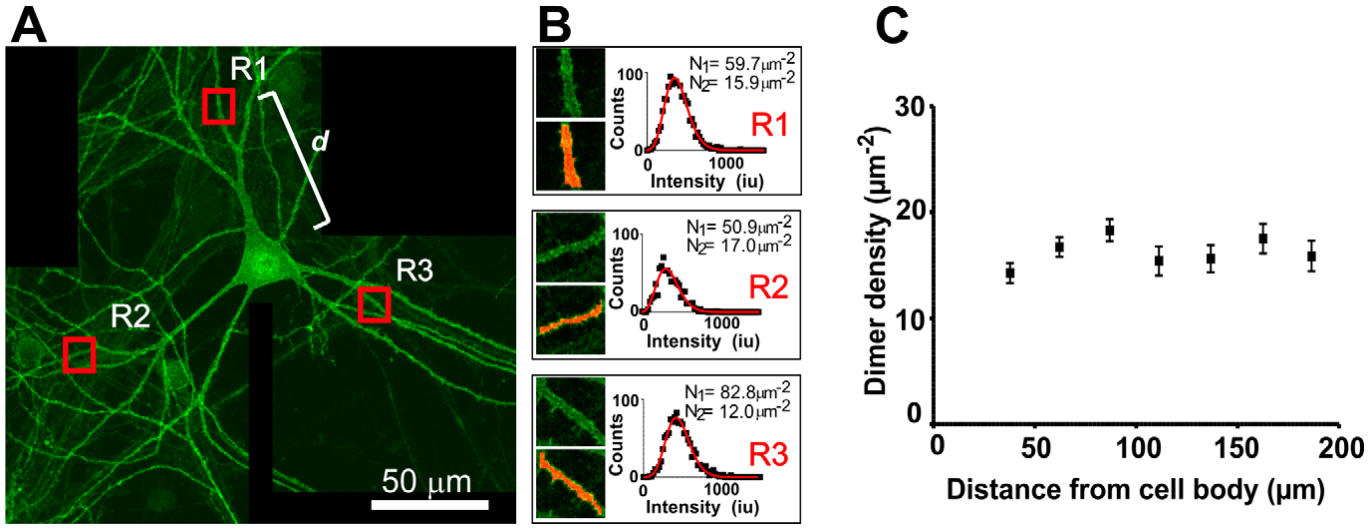
The density of KCC2 is constant along dendrites of neurons in culture. **A**. Three confocal images tiled showing a neuron immunostained with a fluorescent anti-KCC2 antibody Images are of size 1024×1024pixels with a pixel size of 0.115 µm and a 9.1 µs pixel dwell time. Three red squares representing example regions analyzed are shown, in **B**, with their corresponding binary mask used to delineate the labeled dendrite. The intensity histograms of the representative subregions delimited in **A** are shown in **B** with their corresponding SpIDA fit and recovered values of monomer (N_1_) and dimer (N_2_) densities. The distance (e.g., *d* in **A**) between the center of the cell body and the center of the analyzed region was also measured. **C**. Graph of the density of KCC2 dimers at the dendrite surface as a function of the distance from the cell body obtained with SpIDA. 823 regions were analyzed from 27 neurons. Error bars show SEM.

Our simulations were based on the assumption of even distribution of KCC2 along the dendrites and this configuration appears to be sufficient to explain the somato-dendritic gradient observed. However, this does not rule out the possibility of a gradient of KCC2 along the dendrites. To test for the presence or absence of such gradient, we sought to perform quantitative fluorescence immunocytochemical analysis of the distribution of KCC2 along dendrites. Measuring KCC2 immunolabeling may not be sufficient, however, to obtain an estimate of the distribution of functional KCC2 because it has recently been suggested that the oligomeric form of KCC2 is the functional one [31], [32]. To specifically measure the density of KCC2 dimers along the dendrites we took advantage of a technique we recently developed, entitled Spatial Intensity Distribution Analysis (SpIDA) which allows quantitative measurement of the density and oligomerization of proteins from conventional laser scanning confocal microscopy analysis of immunocytochemical labeling [33], [34]. We thus applied SpIDA to analysis of of KCC2 immunostaining of dendrites of the neurons used in the pharmacological experiments described above (Fig. 4). The monomeric quantal brightness was estimated using immunolabeling of KCC2 in neurons that have been in culture for only 5 days, because, at that stage of development, KCC2 has been shown to be essentially monomeric [31]. The monomeric quantal brightness was estimated to be 3.9×10^6^±0.2 (mean ± SEM) intensity units or 3.9±0.2 Miu, and was constant along the dendrite of 5-DIV neurons (52 regions from 11 neurons). Using automated intensity binary masks [35], the dendrites of the mature neurons (> 21 DIV) were carefully detected and intensity histograms were generated for each analyzed region and a two-population (monomers and dimers) mixture model was assumed. For each analyzed region, SpIDA was performed on the image of the z-stack (0.5 µm between images) that had the brightest mean intensity in the chosen region. To estimate the true membrane density of KCC2, the final value for each region was averaged over the two adjacent images of the z-stack. A neuron with example regions and their corresponding histogram and SpIDA fit values are presented in Figure 5A,B. The results indicate that the membrane density of KCC2 is constant along the dendrites, at least as far as 200 µm from the center of the cell body (Fig. 5C).

**Figure 6:**
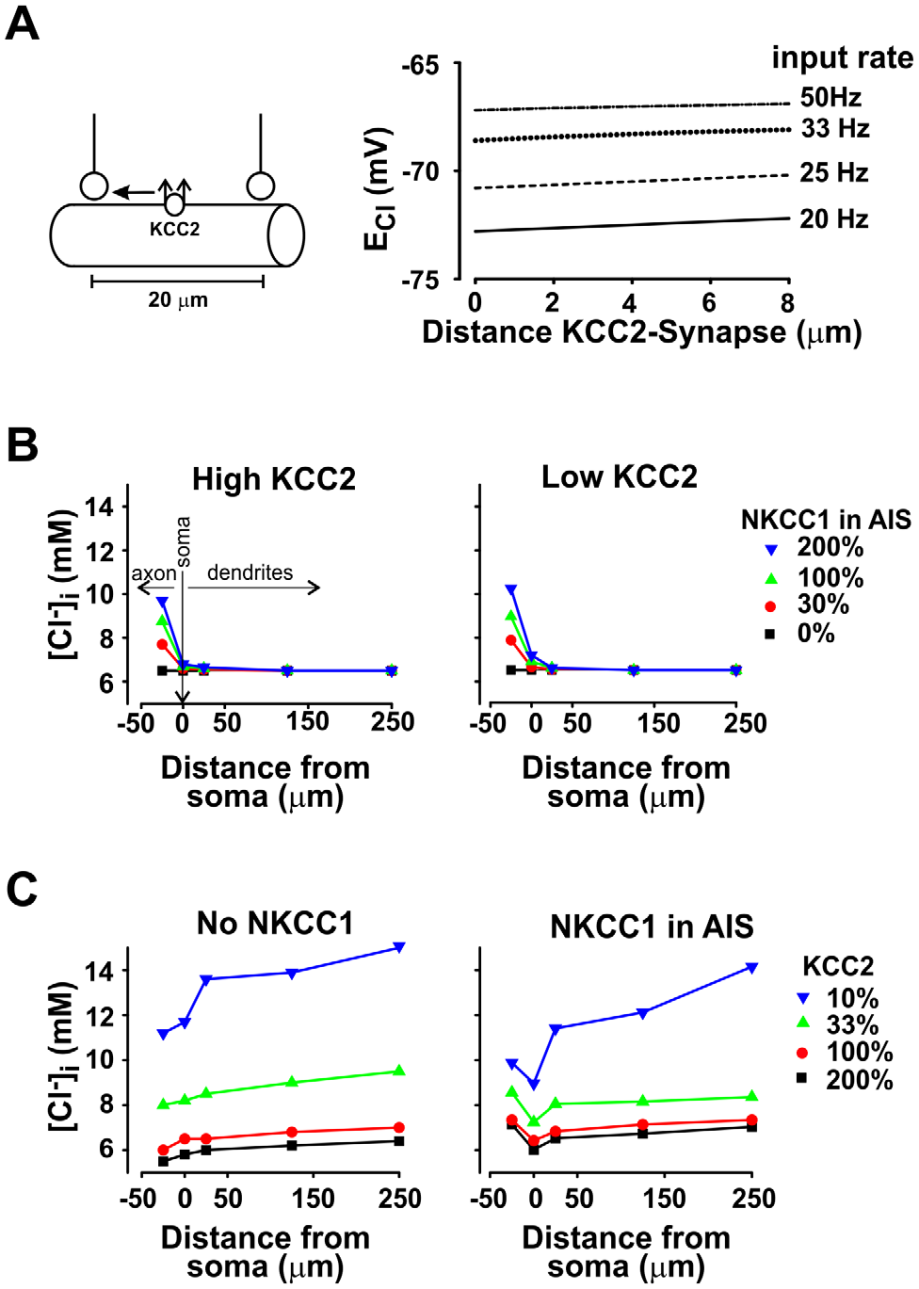
Inhomogenous CCC distribution can create large-scale, but not fine-scale, intracellular [Cl^−^]_i_ gradients. **A.** *left*: To investigate whether the perisynaptic distribution of KCC2 can produce fine-scale intracellular [Cl^−^]_i_ gradients, we varied the subcompartmental distribution of KCC2 by concentrating it in a single location in each compartment at varying distances from a bursting synapse. We divided the compartment into 20 1-µm-long sections. Total amount of KCC2 per compartment was constant at 100%. Inhibitory synapses were located at 20 µm from each other and were activated at high frequency. *Right*: Results show that the subcompartmental distribution has little impact on the perisynaptic value of *E*_Cl,_ which contrasts with the impact of high frequency synaptic input (see Fig. 3) but is consistent with diffusion being responsible for rapid redistribution of intracellular Cl^−^ load. **B.** In the absence of synaptic activity, we inserted different levels of NKCC1 activity in the axon initial segment (AIS) and monitored the axo-somato-dentritic [Cl^−^]_i_ gradient for high (100%) and low (33%) levels of KCC2 activity (uniformly distributed, except in the AIS). Soma corresponds to 0 on *x*-axis; positive distance extends towards dendrites and negative distance extends towards axon, as summarized on left panel. **C.** In the presence of background synaptic activity (*f*_inh_  =  0.4 Hz; *f*_exc_  =  0.1 Hz) we simulated different levels of KCC2 activity (uniformly distributed, except in the AIS) and monitored the axo-somato-dentritic [Cl^−^]_i_ gradient in the presence (100%) or absence of NKCC1 in the AIS.

While our experimental results indicate homogeneous distribution along the dendrite length, this does not necessarily apply to all conditions and, in particular, our analysis did not focus on local inhomogeneities, e.g. microdomains. We therefore also sought to determine if longitudinal intracellular Cl^−^ gradients could also arise from inhomogenous CCC activity at small length scales. For instance, non-uniform distribution of KCC2 at the subcompartent-level might produce local gradients comparable to those observed with synaptic inputs (see Fig. 3). Indeed, clustering of KCC2 has been observed near some synapses [36], but KCC2 near excitatory synapses has been shown to serve a role in scaffolding rather than as a co-transporter [37]. Nevertheless, to test whether subcellular distribution of KCC2 can yield local gradients, we simulated high frequency synapses at 20 µm intervals, between each firing synapses Cl^−^ extrusion through KCC2 was localized at a single point that was placed at different distances from the synapses (Fig. 6A). In all cases, the location of KCC2 had an impact on *E*_Cl_ of <2 mV. Thus, our simulations showed that subcompartemental distribution of KCC2 (i.e. inhomogeneities on the spatial scale of 0-10 µm) has little impact on the perisynaptic value of *E*_Cl_.

The results above do not rule out the possibility of inhomogeneities in CCC expression underlying gradients in other cells types, as well as inhomogeneities in the axon initial segment and soma with respect to dendrites. For instance, absence of KCC2 in the axon initial segment (AIS) [9], [38], selective expression of the inward Cl^−^ transporter NKCC1 in the AIS [28], or the combination of both expression patterns would be expected to cause *E*_Cl_ to be less negative in the AIS. To test *E*_Cl_ in the AIS and how it impacts neighboring compartment, we simulated different levels of NKCC1 in the AIS in combination with different levels of KCC2 in the soma and dendrites with or without background synaptic input (Fig. 6B–C). NKCC1 expression in the AIS can produce an axo-somatic [Cl^−^]_i_ gradient, but this gradient does not extend far, if at all, into the dendrites (Fig. 6B). As expected, combining NKCC1 expression in the AIS with synaptic noise (like in Fig. 4A) resulted in a “double gradient” (Fig. 6C *right panel*).

Thus, simulations in our electrodiffusion model demonstrated that subcellular distribution of GABA_A_R input and CCC activity can produce spatial inhomogeneities in *E*_Cl_, which should translate into inhibitory input having differing efficacy depending on the location of the synapse. This is true even if KCC2 activity is uniformly distributed in the presence of background GABA_A_R input. Moreover, focal Cl^−^ influx through one synapse (or a cluster of synapses) can affect the efficacy of neighbouring synapses, although this depends on subcellular localization of those interacting synapses, *e.g.* proximity to the soma. In contrast, subcompartmental inhomogeneity in KCC2 activity is not sufficient to cause local [Cl^−^]_i_ gradients.

### Diffusion and KCC2 activity determine how robustly the transmembrane chloride gradient is maintained during high frequency synaptic input

**Figure 7:**
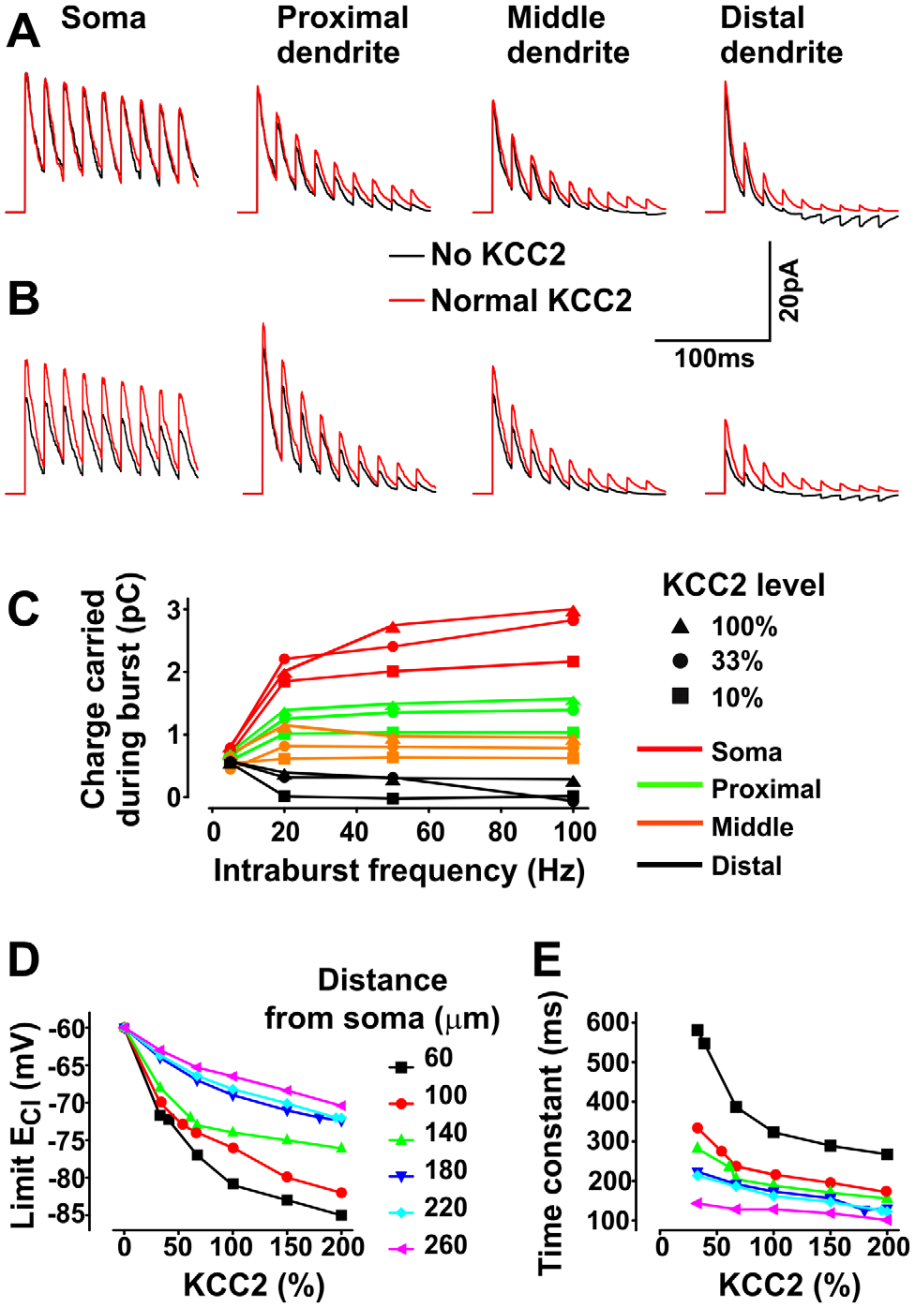
Dependency of Cl^−^ accumulation on the site of synaptic input and KCC2 level. Trains (40 Hz) of inhibitory postsynaptic currents (IPSCs) at a synapse located at one of four positions: soma and proximal, middle, and distal dendrites (40, 100, and 240 µm from soma, respectively) in simulations without (**A**) and with (**B**) background synaptic input (*f*_inh_  =  0.4 Hz, *f*_exc_  =  0.1 Hz). For this set of simulations, a single dendrite was lengthened (and number of compartments increased to 60) relative to the cell geometry summarized in Fig. 1A. Inversion of the IPSC was evident in the distal dendrites under conditions without KCC2 (*right panels*). **C**. Mean intraburst IPSC became smaller (*i.e.* less hyperpolarizing) with increasing distance from the soma and with decreasing KCC2 level. Synaptic background activity was the same as in **B**. Mean IPSC was measured at a “test” synapse activated at 40 Hz for 200 ms every second over 50 s of simulated time. Steady state value of *E*_Cl_ (**D**) and rate at which *E*_Cl_ approaches steady state (**E**) for different KCC2 levels and distances of “test” synapse from the soma. Steady state *E*_Cl_ reported in **D** was measured as the value to which *E*_Cl_ converged when GABA_A_R at the test synapse were artificially held open. This convergence was fit with a single exponential to determine the time constant reported in **E**.

Figures 4 and 6 emphasized how spatial variations in [Cl^−^]_i_ can arise from ongoing GABA_A_R input. To extend these results to include temporal changes in *E*_Cl_, we considered how [Cl^−^]_i_ evolves during stimulus transients. This was motivated by experimental observations that *E*_GABA_ can rapidly collapse during bursts of GABA_A_R synaptic events [20], [28], [39], [40]. Activity-dependent changes in *E*_GABA_ depend on the location of the input: somatic input has less impact on *E*_GABA_ than dendritic input [4], [28]. Simulations in our electrodiffusion model replicated those experimental data (Fig. 7A) as well as results from simpler models [19]. A train of synaptic inputs to the soma produced a small depolarizing shift in *E*_GABA_, which translated into a small reduction in GABA_A_R-mediated current. The depolarizing shift in *E*_GABA_ was greater and occurred increasingly faster for input to progressively more distal dendrites. This was despite the presence of KCC2 (*red*). Removing KCC2 (*black*) increased the amplitude and speed of the collapse in Cl^−^ gradient during high frequency input to distal dendrites, but had virtually no impact for input to the soma. The finding that amplitude of the initial synaptic event in each of the compartments was unaffected by removing KCC2 appears to contradict the observation that the standing [Cl^−^]_i_ gradient depends on KCC2 activity (see Fig. 4). We hypothesized that this was due to the absence of ongoing Cl^−^ load caused by the lack of background synaptic activity. We therefore repeated simulations shown in Figure 7A but with background synaptic input (Fig. 7B). As predicted, the initial IPSC amplitude was affected by the KCC2 activity level when background synaptic input was present (compare Fig. 7B and A). These results suggest that the rate of local intracellular Cl^−^ accumulation depends principally on diffusion (which redistributes the intracellular Cl^−^ load), whereas the extent of accumulation depends on KCC2 activity (which reduces intracellular Cl^−^ load via extrusion).

To investigate these processes more thoroughly, we systematically varied the intraburst frequency, location of the “test” synapse and KCC2 activity, and we measured the mean IPSC amplitude at the “test” synapse throughout the burst. During high frequency input to distal dendrites, the net mean current through GABA_A_R synapses switched from outward to inward whereas the same rate of input to the soma continued to produce strong outward currents (Fig. 7C). Thus, while increasing intraburst frequency can effectively enhance hyperpolarization in the soma, it rapidly becomes ineffective in dendrites and can even become depolarizing in distal dendrites. For a fixed intraburst frequency, *E*_Cl_ converged to different steady-state levels (Fig. 7D) with different rates (Fig. 7E) depending on the location of the test synapse and the level of KCC2 activity. In other words, the steady-state value of [Cl^−^]_i_ increased with distance from the soma (reminiscent of the standing gradient reported in Fig. 4A and C) and it decreased when KCC2 activity was increased. On the other hand, Cl^−^ accumulation converged to a steady state more rapidly with increased KCC2 activity as well as with distance from the soma. The two convergence processes are due to different phenomena: Enhanced KCC2 activity allows the dendrite to restrict the extent of Cl^−^ accumulation (see above), while Cl^−^ accumulates faster in distal dendrites simply because the effective volume is smaller and diffusion is restricted. In summary, under dynamic conditions, restricted diffusion in distal dendrites causes a rapid collapse of *E*_GABA_, but the extent of this collapse is limited by KCC2, consistent with experimental measurements [8], [9], [28].

### In dendrites, distributed GABA_A_R input mediates greater inhibition than higher frequency focal input

**Figure 8:**
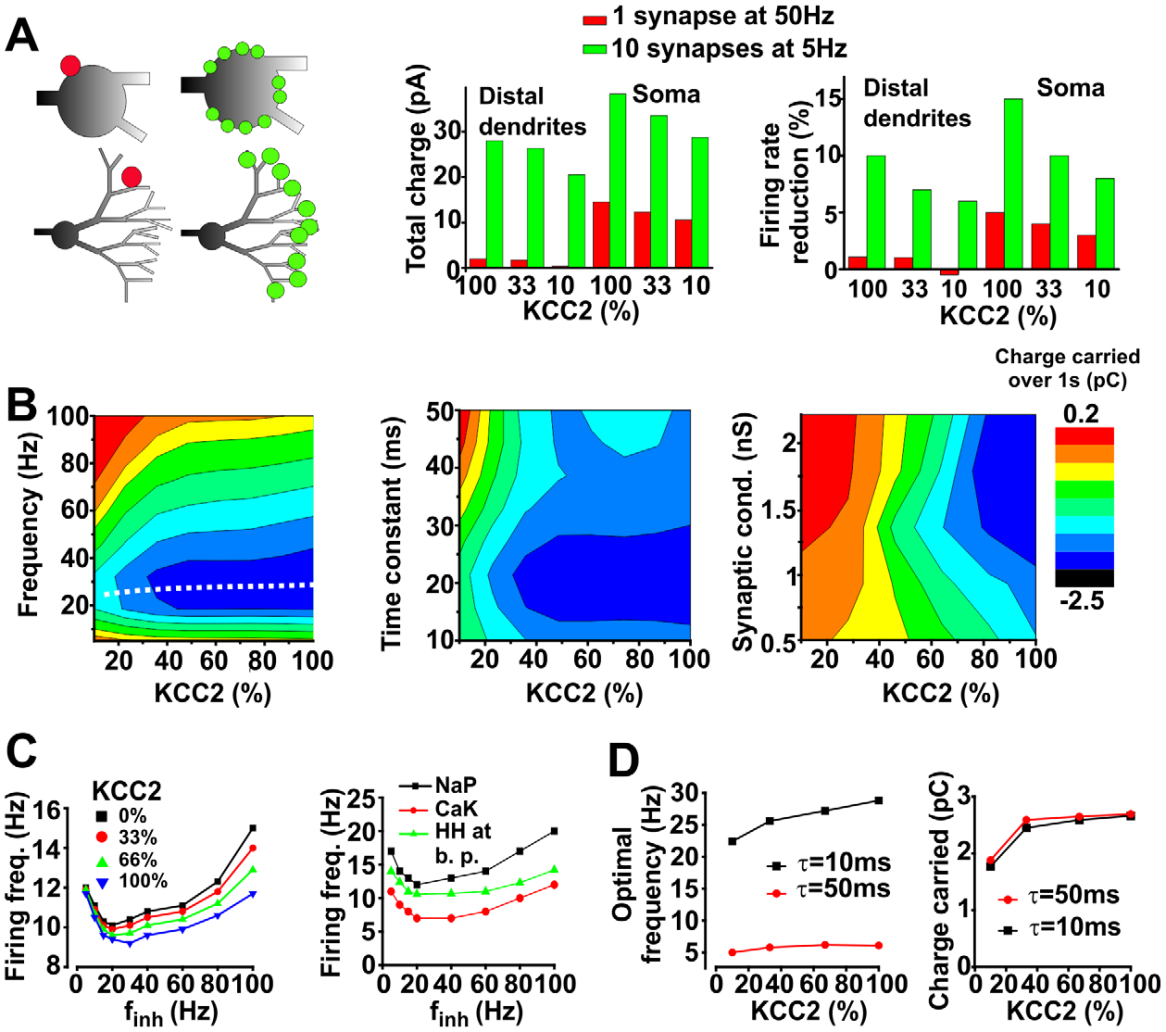
Efficacy of inhibition depends on spatial and temporal features of GABA_A_R input. **A.** Schematic shows synapse positioning (*left panel*). GABA_A_R input clustered at a single synapse (*red*) produced less outward current than the same total input distributed across ten spatially separated synapses (*green*), especially for input to the distal dendrites (*center panel*). To ensure that “total charge” translates into functionally relevant inhibition (*i.e.* reduction in spiking), we submitted the model to distributed excitatory input (*f*_exc_  =  0.2 Hz) and measured firing rate. As expected, reduction in firing frequency was greater when inhibitory input was spatially distributed (*right panel*). **B.** Net charge carried through a “test” synapse (*color*) consistently decreased as KCC2 activity was reduced, but increasing the frequency (*left panel*), time constant (*middle panel*) or conductance (*right panel*) of input at that synapse did not necessarily increase current amplitude. For the *left panel*, the time constant was held at 10 ms while the input frequency and KCC2 level were varied; the dotted line shows optimal frequency, which is re-plotted in **D**. For the *middle panel*, the input frequency was held at 30 Hz while the time constant and KCC2 level were varied. For the *right panel* input frequency and time constant were held at 30 Hz and 10 ms respectively while the conductance and KCC2 level were varied. Background synaptic activity was included in these simulations (*f*_inh_  =  0.4 Hz, *f*_exc_  =  0.1 Hz). Test synapse was positioned at 50 µm from the soma. **C**. We performed simulations similar to that in **B** but added distributed excitatory input to assess inhibition on the basis of firing rate reduction rather than on the basis of total charge (*left panel*). The pattern of inverted bell-shaped curves is consistent with **B**, thus confirming a net change in inhibition at the whole cell level. The graph on the *right* illustrates results obtained from simulations with models including Ca^2+^-activated K^+^ channels or persistent Na^+^ channels. We also concentrated dendritic HH channels at branch points while preserving the total conductance of these channels. Results were qualitatively the same as in the graph on the *left*. **D.** Optimal input frequency depending on KCC2 level and time constant (*left panel*) and the corresponding current (*right panel*). Black curves correspond to dotted line on left panel of **B**. Note that this is the optimal frequency for activation of a single “test” synapse; optimal input frequency would necessarily decrease as the number of activated synapses increased, although the exact relationship would depend on the spatial distribution of those active synapses (see **A**) as well as the level of background synaptic activity.

The above results led us to predict that, for equivalent total synaptic input, many broadly distributed GABA_A_R synapses activated at low frequency would produce greater hyperpolarization than a few clustered synapses (or just one synapse) activated at higher frequency, especially for synapses located on distal dendrites. We tested this by comparing the outward current produced by one synapse activated at an intraburst frequency of 50 Hz with the total hyperpolarizing current produced by ten distributed synapses activated at 5 Hz; this was repeated for dendritically and somatically positioned synapses (Fig. 8A). In the soma, ten synapses activated at 5 Hz produced more outward current than one synapse activated at 50 Hz (Fig. 8A *middle*). This is due to the fact that the total synaptic conductance does not scale linearly with frequency because of saturation. Even more important is the fact that distributed dendritic input is capable of producing a strong outward current despite Cl^−^ accumulation, whereas clustered dendritic input was totally inefficient in producing an outward current. These results suggest that dendritic inhibition is most effective when spatially distributed, consistent with data in Figs. 3 and 6. Maintaining spatially distributed GABA_A_ synapses in dendrites is also important because the rapid dynamic collapse of distal hyperpolarizing GABA_A_R currents will limit their effectiveness at controlling somatic signals because membrane potential changes extend farther than changes in conductance [8], [41]. Given that shunting remains even when *E*_Cl_ collapses, we submitted the neuron to distributed excitatory input and measured the mean firing frequency of the model neuron to verify that loss of hyperpolarizing current translates into effective disinhibition (Fig. 8A *right*). We found that firing rate reduction mirrored the change in charge carried (cf. Fig. 8A *right* and *middle* panels).

### Enhancing GABA_A_R input may fail to enhance inhibition under conditions of impaired chloride homeostasis

In addition to synapse location, the rate and duration of synaptic inputs would be expected to interact with dynamic changes in *E*_GABA_ to alter the efficacy of inhibition. Although increasing the rate or duration of GABA_A_R inputs may initially increase IPSC amplitude, such changes would also accelerate depletion of the Cl^−^ gradient and thereby eventually reduce IPSC amplitude, at least when Cl^−^ influx overwhelms local diffusion mechanisms and Cl^−^ extrusion capacity. Using our model, we studied the influence of KCC2 activity level, synaptic frequency and time constant of GABA_A_R-mediated events (τ_IPSC_) on the mean current through a dendritic GABA_A_R synapse. Simulations indicated that increasing KCC2 activity always led to larger mean outward current. In contrast, increasing synaptic input frequency (Fig. 8B *left*) or τ_IPSC_ (Fig. 8B *right*) did not necessarily increase the mean current; in both cases, the mean current was largest at intermediate values of those parameters. Similarly, mean firing rate was reduced most at intermediate values of those parameters (Fig. 8C). To establish the generality and robustness of the result, we repeated simulations for neurons endowed with different ion channels affecting spike generation. We added non-inactivating Ca^2+^-activated K^+^ channels known to decrease firing rate or persistent Na^+^ channels known to increase firing rate, and we also performed simulations in which dendritic Hodgkin-Huxley (HH) channels were concentrated at branch points. Although these modifications to the model changed the overall firing rate, our qualitative finding remained unchanged; that is, firing rate increased if GABA_A_R input was augmented beyond a certain level (Fig. 8C *right*).

The above results indicate that more or longer GABA_A_R inputs may not always produce more inhibition, *i.e.* stronger outward current. We therefore asked what GABA_A_R input conditions produce the strongest inhibition? This question was addressed by measuring which parameter combinations produced the largest outward current. We found that the GABA_A_R input frequency yielding the largest outward current increased with KCC2 activity and decreased with τ_IPSC_ (Fig. 8D). This optimal frequency was as low as 6 Hz when KCC2 activity was depleted to 10% of its normal value and τ_IPSC_ was set to 50 ms; in other words, GABA_A_R-mediated synaptic events occurring either at lower or at higher frequencies than 6 Hz produced less outward current. The optimal GABA_A_R input frequency climbed to 28 Hz when KCC2 activity was set to baseline and τ_IPSC_ was set to 10 ms. Thus, the optimal GABA_A_R input frequency may vary quite widely depending on other factors, but the key observation is that beyond some point (determined by the robustness of Cl^−^ homeostasis), more GABA_A_R input does not necessarily produce more inhibition. Increasing the frequency of GABA_A_R input showed a similar inverted bell-shaped curve when estimating effective inhibition with either total charge carried or firing rate reduction (Fig. 8B and C).

### Net current through GABA_A_R depends on the balance of chloride and bicarbonate flux

**Figure 9:**
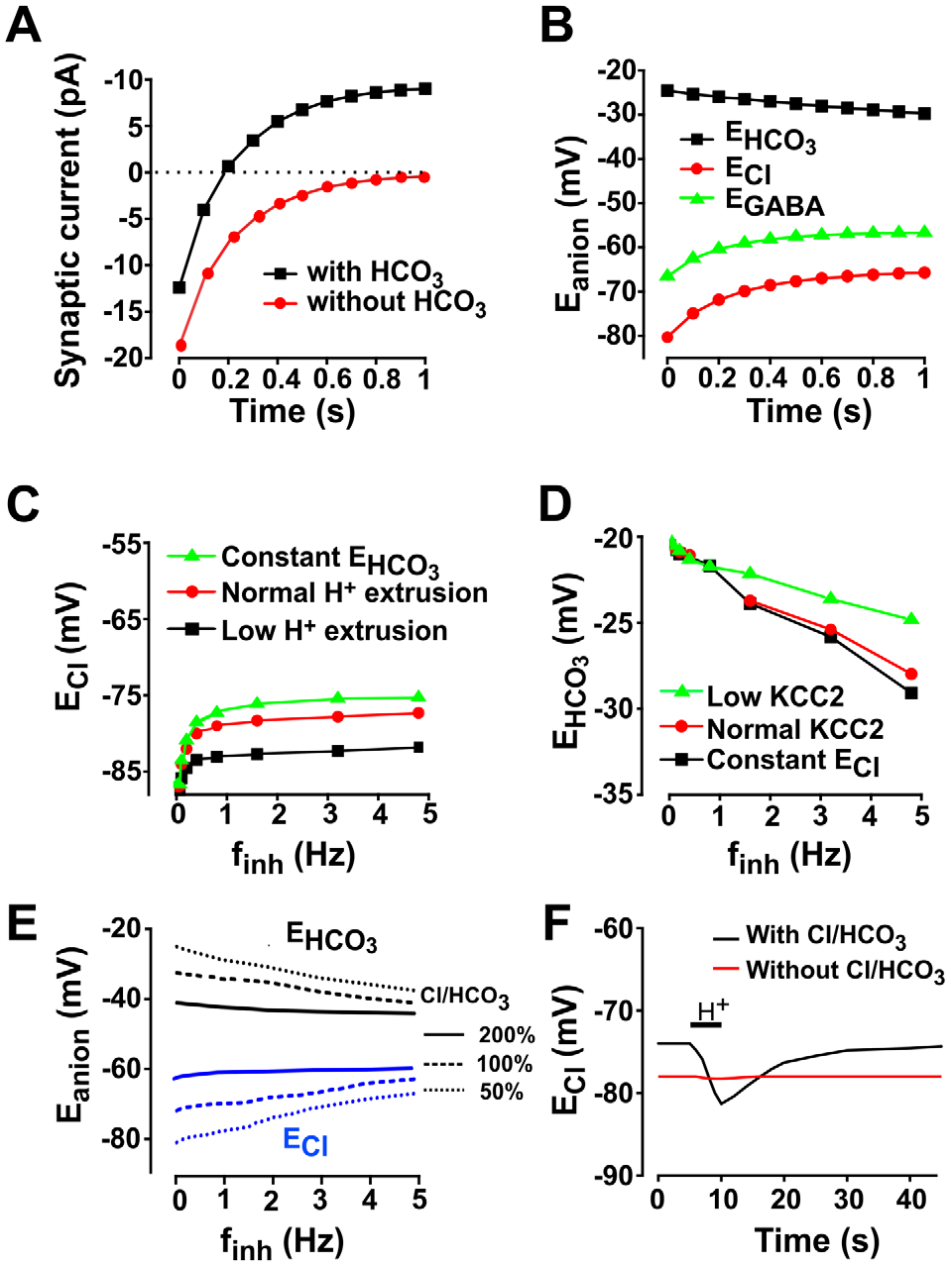
Trade-off between robustness of HCO_3_^−^ and Cl^−^ homeostasis. **A.** Change in synaptic current over time as GABA_A_R synapse is held open. Notice in the standard model (*black*) that current eventually inverted; in contrast, current decayed to zero but did not invert in the model without HCO_3_^−^ efflux (*red*). **B.** Change in reversal potentials over time for standard model and same test conditions as in **A**. Although small compared to changes in *E*_Cl_, *E*_HCO3_ did shift (in the opposite direction). The balance of those changes determines the net shift in *E*_GABA_, which explains the functional implications of predictions tested in **C** and **D** – a reduced change in *E*_HCO3_ should produce an enhanced change in *E*_Cl_, whereas a reduced change in *E*_Cl_ should produce an enhanced change in *E*_HCO3_. **C.** Encouraging HCO_3_^−^ efflux through GABA_A_R by holding [HCO_3_^−^] constant (*green*) exacerbated the depolarizing shift in *E*_Cl_. Discouraging HCO_3_^−^ efflux by reducing H^+^ extrusion to 33% of normal (black), which in turn discourages the forward reaction catalyzed by carbonic anhydrase and accelerates depletion of intracellular HCO_3_^−^, mitigated the depolarizing shift in *E*_Cl_. **D.** Conversely, encouraging Cl^−^ influx through GABA_A_R by holding [Cl^−^] constant (*black*) exacerbated the hyperpolarizing shift in *E*_HCO3_. Discouraging Cl^−^ influx by reducing KCC2 activity to 10% of normal (green) mitigated the hyperpolarizing shift in *E*_HCO3_. Effects in **C** were stronger than those in **D**, which illustrates how inter-relationships can be asymmetrical, *i.e.* pH regulation has a stronger impact on [Cl^−^] dynamics than Cl^−^ regulation has on pH dynamics under the conditions simulated here. **E.** Simulations similar to the ones conducted in **C** and **D** were performed but with the addition of Cl^−^/HCO_3_^−^ exchanger at different levels of activity. **F.** We performed a simulation in which we added an artificial H^+^ influx for 5 s (horizontal bar). The proton influx caused a sizeable drop in [HCO_3_^−^]_i_, thereby producing a hyperpolarizing shift in *E*_HCO3_; that shift is greater in the model without the Cl^−^/HCO_3_^−^ exchanger (not shown). The resulting change in HCO_3_^−^ gradient caused an inversion of Cl^−^/HCO_3_^−^ exchange that led to a significant lowering of *E*_Cl_; this did not occur in the absence of the Cl^−^/HCO_3_^−^ exchanger.

Results of simulations presented in Figure 7 showed that the current through GABA_A_R could reverse polarity if there was sufficient accumulation of intracellular Cl^−^. However, as the Cl^−^ gradient collapses, one would expect Cl^−^ flux to stop, but not to change its direction; likewise, the IPSCs would be expected to become smaller but not to invert. Indeed, if the GABA_A_R is modeled as passing only Cl^−^ ions, the IPSC decreases in size as Cl^−^ accumulates intracellularly, but it does not reverse direction (Fig. 9A) thus showing that bicarbonate flux must be accounted for in order to explain IPSC inversion [42], [43]. An important and novel feature of our model is that HCO_3_^−^ is not assumed to be constant. Even if the relative stability of [HCO_3_^−^]_i_ has been shown to result from complex interaction between HCO_3_^−^ efflux, carbonic anhydrase-mediated reaction and proton extrusion mechanisms, most models choose to consider it constant *de facto*. However, simulating the various mechanisms involved in [HCO_3_^−^]_i_ management proved a useful tool for investigating the legitimacy of assuming [HCO_3_^−^]_i_ is constant and for studying potential interactions between Cl^−^ and HCO_3_^−^ dynamics. Bicarbonate efflux produces an inward current, but that current is (normally) masked by the larger outward current produced by Cl^−^ influx, since the permeability ratio between Cl^−^ and HCO_3_^−^ anions is approximately 4∶1 [2], [43]. But as the Cl^−^-mediated outward current becomes smaller, the HCO_3_^−^-mediated inward current becomes *relatively* larger, eventually causing the net current through GABA_A_R to become inward. Unlike the Cl^−^ gradient, the HCO_3_^−^ gradient tends not to collapse (Fig. 9B) because intracellular HCO_3_^−^ is replenished by carbonic anhydrase-catalyzed conversion of CO_2_, which can readily diffuse across the membrane [44], [45].

But although the reactants of the carbonic anhydrase-catalyzed reaction (*i.e.* CO_2_ and H_2_O) are not depleted, the forward reaction produces H^+^ in addition to HCO_3_^−^. By removing HCO_3_^−^, GABA_A_R activity would be expected to reduce the intracellular pH, which has been observed experimentally [24]. Since accumulation of intracellular H^+^ shifts the equilibrium point of the reaction, intracellular HCO_3_^−^ slowly decreases, with a time constant in the order of several seconds, which explains the small hyperpolarizing shift in *E*_HCO3_ seen in Figure 9B over long time scales. By *E*_Cl_ and *E*_HCO3_ shifting in opposite directions, *E*_GABA_ tends toward the membrane potential. We therefore predicted that reducing changes in *E*_HCO3_ would lead to greater changes in *E*_Cl_ and, vice versa, that reducing changes in *E*_Cl_ would lead to greater changes in *E*_HCO3_. To test the first prediction, [HCO_3_^−^]_i_ was held constant (thus maintaining HCO_3_^−^ efflux), which enhanced the depolarizing shift in *E*_Cl_; on the other hand, increasing intracellular HCO_3_^−^ depletion by reducing proton extrusion via the Na^−^-H^+^ exchanger (thus reducing HCO_3_^−^ efflux) mitigated the depolarizing shift in *E*_Cl_ (Fig. 9C). To test the second prediction, [Cl^−^]_i_ was held artificially constant, which enhanced the hyperpolarizing shift in *E*_HCO3_; conversely, increasing intracellular Cl^−^ accumulation by reducing Cl^−^ extrusion via KCC2 mitigated the hyperpolarizing shift in *E*_HCO3_ (Fig. 9D). These results demonstrate a trade-off between stability of [Cl^−^]_i_ and stability of intracellular pH based on their common reliance on [HCO_3_^−^]_i_. It remains an open question whether [Cl^−^]_i_ or intracellular pH is more strongly regulated under normal conditions, but one can reasonably extrapolate when KCC2 activity is reduced, that the primary depolarizing shift in *E*_Cl_ will conspire with a smaller secondary hyperpolarizing shift in *E*_HCO3_ to produce a large depolarizing shift in *E*_GABA_. This is particularly relevant to steady state conditions because, on the time scale of individual synaptic events, pH buffering mechanisms are not saturated, while on longer time scales, the rate limiting components of HCO_3_^−^ homeostasis are the slower kinetics of the HCO_3_^−^ and H^+^ membrane transporters.

The Cl^−^/HCO_3_^−^ exchanger can also play a role in pH management and Cl^−^ homeostasis regulation. To gain some insight into the impact of this exchanger, we repeated simulations of Figure 9C–D adding different levels of Cl^−^/HCO_3_^−^ exchanger activity to the model. As is the case for such ion exchangers, the Cl^−^/HCO_3_^−^ exchanger will drive *E*_Cl_ and *E*_HCO3_ towards one another, namely depolarizing *E*_Cl_ and hyperpolarizing *E*_HCO3_ (Fig. 9E). This result may seem counterintuitive since the exchanger would be expected to play a helpful role in pH management. However, in the instance of another source of acidification, *E*_HCO3_ can undergo a hyperpolarizing shift, and the resultant change in HCO_3_^−^ gradient can reverse Cl^−^/HCO_3_^−^ transport, driving Cl^−^ out and HCO_3_^−^ in, thus preventing overt acidification (Fig. 9F).

These results predict that *E*_Cl_ can become more hyperpolarized during episodes of acidification. To test this, we modeled H^+^ influx occurring over 5 seconds and monitored the time course of *E*_Cl_ during and after acidification in simulations with and without the Cl^−^/HCO_3_^−^ exchanger. In such simulations, proton influx triggers a reaction with HCO_3_^−^ thus leading to a decrease in [HCO_3_^−^]_i_. In turn, this leads to hyperpolarization of *E*_HCO3_ which will eventually become more hyperpolarized than *E*_Cl_, effectively inverting the exchanger and leading to hyperpolarization of *E*_Cl_ (Fig. 9F). As the influx of H^+^ is stopped, H^+^ extrusion through the Na^+^/H^+^ exchange restores pH and the carbonic anhydrase mediated reaction is able to replenish intracellular HCO_3_^−^. As this slow change in [HCO_3_^−^]_i_ translates into a change in the activity of the Cl^−^/HCO_3_^−^ exchanger, the value of *E*_Cl_ slowly becomes more depolarized until it returns to its resting value (Fig. 9F). As expected, these changes in *E*_Cl_ cannot be observed when simulations are conducted without the Cl^−^/HCO_3_^−^ exchanger (Fig. 9F). Thus, the Cl^−^/HCO_3_^−^ exchanger may be seen as a failsafe mechanism preventing overt acidification, at least when this acidification is not caused by HCO_3_^−^ efflux through GABA_A_ channels.

### Accumulation of extracellular potassium influences GABA_A_R-mediated current via a multi-step feedback loop

**Figure 10:**
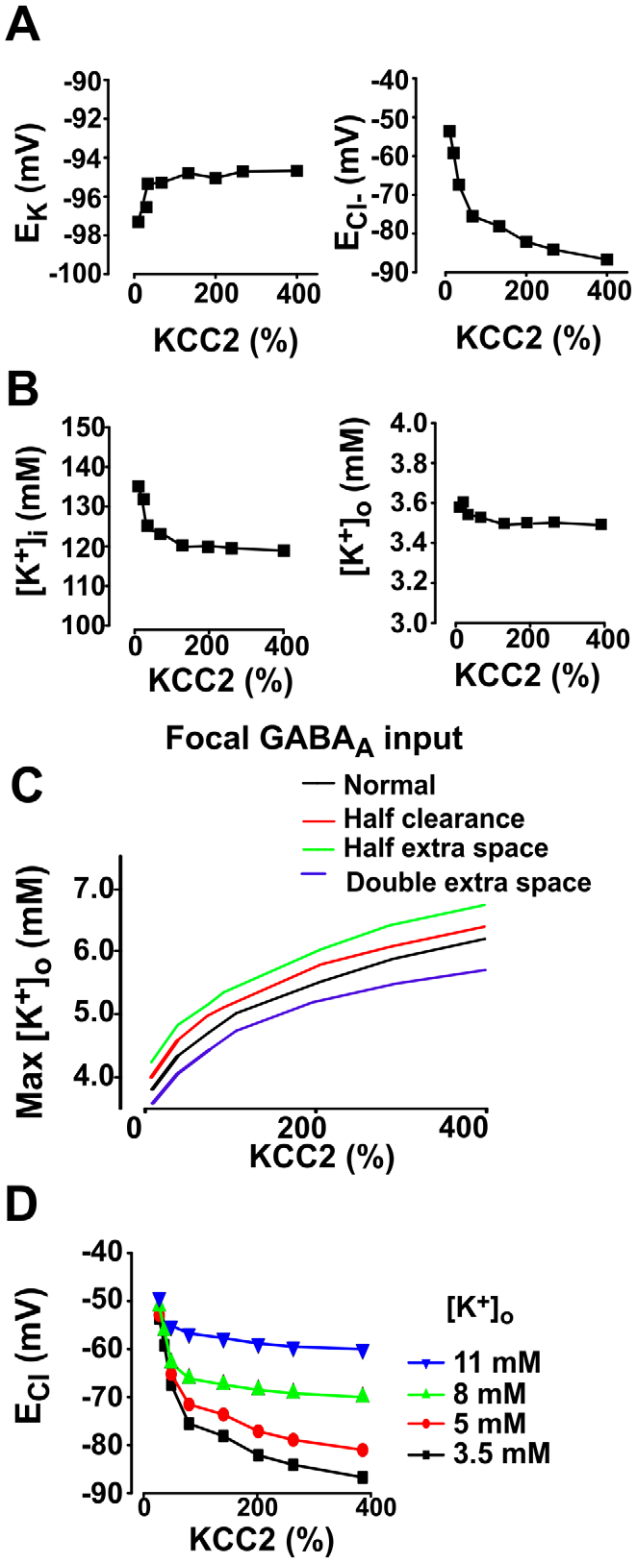
Interactions between [Cl^−^] regulation and [K^+^] regulation. **A.** Variation of KCC2 levels caused sizeable shifts in *E*_Cl_ (*right panel*) but had negligible effects on *E*_K_ (*left panel*). Background synaptic activity was *f*_exc_  =  0.2 Hz and *f*_inh_  =  0.8 Hz. **B.** Intra- and extracellular concentrations of K^+^ for same simulations reported in **A**. Although extracellular K^+^ levels are low, [K^+^]_o_ remains relatively stable due to other mechanisms, *e.g.* extracellular diffusion. This explains why *E*_K_ remains relatively constant in **A**. **C.** Maximal [K^+^]_o_ reached by applying a 500 nS GABA conductance to a dendrite. Time constant for diffusion from the FH space was tested at 100 and 200 ms (which corresponds to normal and 50% slower extracellular K^+^ clearance) as well as with variable extracellular space. **D**. *E*_Cl_ as a function of the mean frequency of inhibitory input for various fixed levels of [K^+^]_o_.

To extrude Cl^−^ from the cell, KCC2 must pass an equal number of K^+^ ions since the net process is electroneutral. Therefore, K^+^ efflux through KCC2 could reduce the transmembrane K^+^ gradient and produce a depolarizing shift in *E*_K_, which would, in turn, reduce Cl^−^ extrusion via KCC2 because of the reduction in KCC2 driving force. To investigate this putative negative feedback mechanism, we varied KCC2 activity and measured the impact on *E*_K_ (measured at the soma) in a model neuron receiving a fixed level of background excitatory and inhibitory synaptic input. Simulations showed that under conditions of distributed GABA_A_R input at *in vivo*-like background frequencies, KCC2 activity actually had little impact on *E*_K_ unlike its large impact on *E*_Cl_ (Fig. 10A, compare *left* and *right* panels). We investigated this further by monitoring intra- and extracellular concentrations of K^+^ (Fig. 10B). Although large in absolute terms, changes [K^+^]_i_ were small in relative terms, yielding much smaller shifts in *E*_K_ than those observed with *E*_Cl_. Furthermore, KCC2 activity had only a small influence on [K^+^]_o_, which is controlled principally by the balance of K^+^ leak conductance, active pumping by the Na^+^-K^+^-ATPase, and extracellular diffusion.

The insignificant effect of KCC2 activity on [K^+^]_o_ is apparently inconsistent with experimental observations [46], but those experiments involved applying a heavy Cl^−^ load, which is not comparable to the physiological conditions tested in Figure 10A and B. To test whether a larger Cl^−^ load could provoke a KCC2-mediated increase in [K^+^]_o_, we simulated a constant 5 nS, 500 ms-long GABA_A_R conductance on a dendrite. Under those conditions, [K^+^]_o_ was significantly altered by KCC2 activity, as shown by the positive correlation between the maximal value of [K^+^]_o_ and KCC2 level (Fig. 10C). Repeating those simulations with reduced extracellular K^+^ clearance confirmed that extracellular diffusion did not dramatically alter [K^+^]_o_ under these “heavy load” conditions (Fig. 10C). Regardless of whether KCC2 activity does or does not influence extracellular K^+^ accumulation, extracellular K^+^ accumulation is nonetheless expected to reduce the efficacy of KCC2 by reducing its driving force. To test this, we repeated the simulations shown in Figure 1D with different fixed values of [K^+^]_o_ and observed that the KCC2 efficacy is indeed reduced by the extracellular K^+^ accumulation and stops passing ions when [K^+^]_o_  =  10 mM (Fig. 10D).

It is important to understand that changes in [K^+^]_o_ have a much larger effect on *E*_K_ than equivalent absolute changes in [K^+^]_i_. Hence, although KCC2 activity is not expected itself to change *E*_K_ under normal physiological conditions (see above), changes in *E*_K_ caused by other factors (*e.g.* high firing rates, reduced Na^+^-K^+^-ATPase activity, etc.) reduce KCC2 activity. In other words, there is no *closed* negative feedback loop directly linking KCC2 and *E*_K_, but extrinsic factors can modulate Cl^−^ extrusion by affecting extracellular K^+^ accumulation. Indeed, it is significant that Cl^−^ extrusion could be reduced (and inhibition thereby rendered ineffective) under conditions where excessive spiking (perhaps the result of disinhibition) causes extracellular K^+^ accumulation – this would constitute a multi-step positive feedback loop (see also below).

### Failure to control spiking increases chloride accumulation through a positive feedback loop that leads to catastrophic failure of inhibition

**Figure 11:**
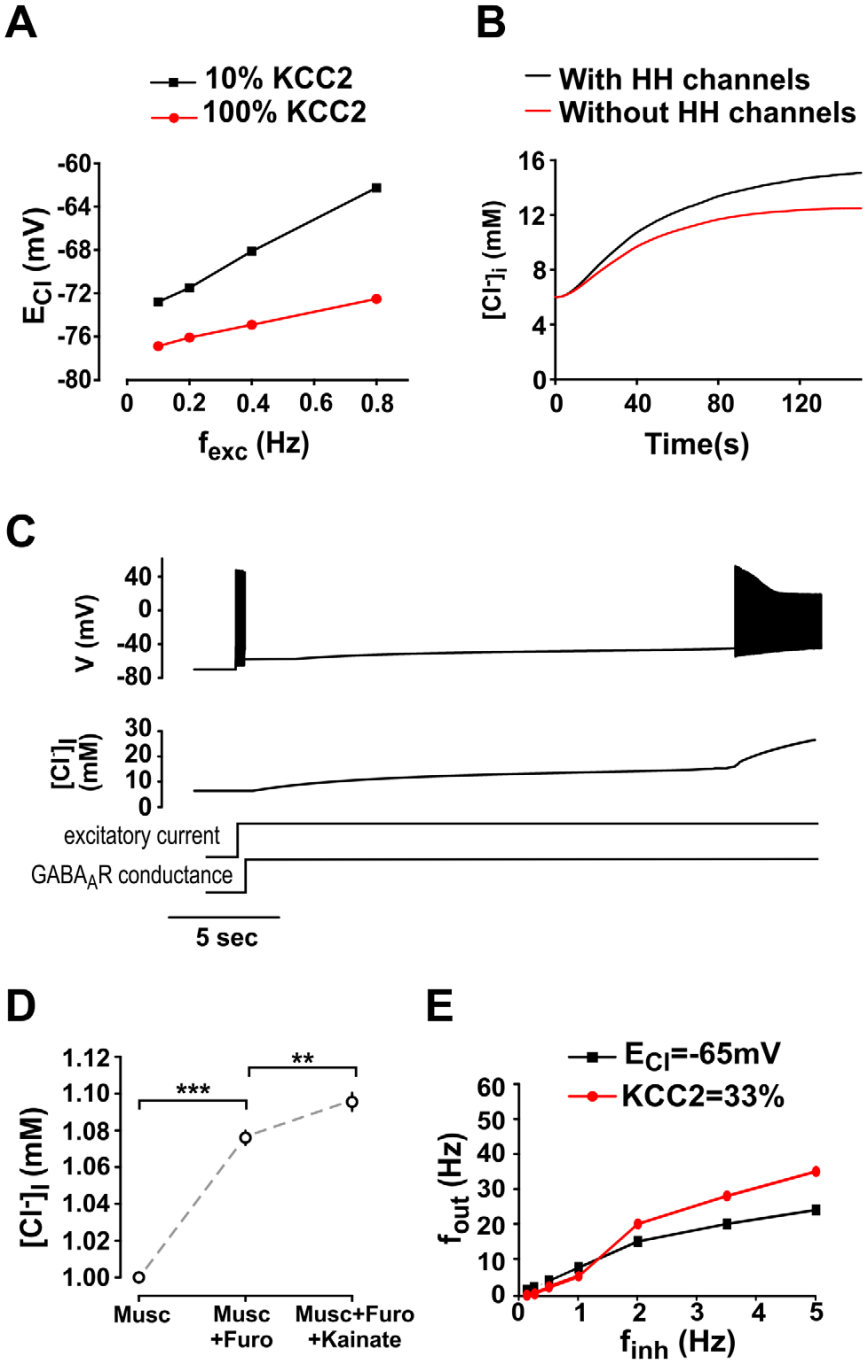
Effects of membrane potential on intracellular Cl^−^ accumulation. **A.** Varying the rate of excitatory synaptic drive (*f*_exc_) caused a depolarizing shift in *E*_Cl_ secondary to changes in average membrane potential. *f*_inh_ was fixed at 0.4 Hz. **B.** Spiking exacerbates intracellular Cl^−^ accumulation as illustrated here by convergence of the model to different steady state [Cl^−^]_i_ depending on whether the model does or does not contain HH channels (*i.e.* does or does not spike, respectively). For this simulation, KCC2 activity was low (10%), *f*_inh_  =  0.8 Hz, and *f*_exc_  =  0.4 Hz. **C.** Sample traces showing inter-relationship between [Cl^−^]_i_ and spiking. Neuron began spiking when constant excitatory current was applied to the soma, but without any concomitant change in [Cl^−^]_i_ since there was not yet any GABA_A_R-mediated conductance. Turning on constant GABA_A_R conductance in the soma terminated spiking, but at the expense of intracellular Cl^−^ accumulation. Chloride slowly accumulated over the next several seconds until membrane potential reached spike threshold, at which point spiking resumed and Cl^−^ began a second phase of accelerated accumulation. **D.** To test whether Cl^−^ accumulation is exacerbated by excitatory synaptic input in real neurons, somatic Cl^−^ concentration was measured using FLIM in neurons with or without glutamatergic receptor activation by kainate. As predicted by simulations, Cl^−^ accumulation was greater in neurons exposed to kainate. Furosemide was applied to block KCC2 activity in these experiments (**, p < 0.001; ***, p < 0.0001). Data from 56 cells from 5 coverslips. **E.** Comparison of input-output curve for static (*black*) vs. dynamic (*red*) *E*_Cl_. Discrepancies between the curves clearly demonstrate that *E*_Cl_ cannot be approximated as constant value when considering a range of input conditions.

As shown in previous sections, GABA_A_R input and KCC2 activity are prominent determinants of *E*_Cl_. However, since Cl^−^ influx depends on the Cl^−^ driving force (*i.e. V* – *E*_Cl_), variation in membrane potential will influence intracellular Cl^−^ accumulation, as shown in voltage clamp experiments [20]. Therefore, we predicted that increased depolarization caused by increased synaptic excitation would exacerbate intracellular Cl^−^ accumulation. To test this, the frequency of inhibitory synaptic events, *f*_inh_, was fixed at 0.4 Hz/synapse while the frequency of excitatory synapses, *f*_exc_, was varied (0.4 Hz was chosen for inhibitory events so that when *f*_exc_/*f*_inh_  =  2, *f*_exc_ was still within its normal physiological range [24], ). As predicted, the depolarizing shift in *E*_Cl_ scaled with *f*_exc_ (Fig. 11A). Moreover, given that spike generation makes membrane potential a highly nonlinear function of synaptic activity, we further predicted that the presence or absence of spiking would have a profound influence on [Cl^−^]_i_ because each spike represents a large, albeit short, increase in Cl^−^ driving force; in other words, if GABA_A_R channels are open during a spike, those spikes are expected to dramatically accelerate intracellular Cl^−^ accumulation. To test this, we measured Cl^−^ accumulation in a model with and without spikes (*i.e.* with and without HH channels, respectively). Results confirmed that Cl^−^ accumulation was indeed increased by spiking (Fig. 11B). The time series in Figure 11C shows the biphasic Cl^−^ accumulation associated with this phenomenon: When inhibition was first “turned on”, it successfully prevented spiking but, over time, [Cl^−^]_i_ increased asymptotically toward some steady-state value. If the associated steady-state *E*_GABA_ was above spiking threshold (as in Fig. 11C), the membrane potential could increase beyond threshold and the neuron began spiking, at which point intracellular Cl^−^ began a second phase of accumulation. This second phase of Cl^−^ accumulation was paralleled by acceleration of the spike rate – clear evidence of the predicted positive feedback loop between spiking and Cl^−^ accumulation, which leads to catastrophic failure of inhibition.

To verify experimentally the model prediction that excitatory activity exacerbates intracellular Cl^−^ accumulation, especially when KCC2 activity is depleted, we performed [Cl^−^]_i_ measurements in primary cultured neurons exposed to muscimol, followed by addition of furosemide and kainate. The latter was to cause tonic activation of AMPA subtype glutamate receptors. As predicted by the model, addition of furosemide caused Cl^−^ accumulation in the cell, and subsequent application of kainate led to further accumulation (Fig. 11D).

The fact that *E*_Cl_ collapses as a result of GABA_A_R activity itself (Figs. 1, 3, 9) as well as excitatory input (Fig. 11A and D) and spiking (Fig. 11B and C) highlights the importance of treating *E*_Cl_ as a dynamic variable. To assess the importance of those dynamics on GABA_A_R modulation of the firing rate, we compared the relationship between firing rate and synaptic input in conditions where both inhibitory and excitatory input change in a proportional manner (*i.e.*, *f*_inh_ α *f*_exc_). We performed simulations in which *E*_Cl_ was treated as a static value (as in conventional cable models) or as a dynamic variable (as in our electrodiffusion model). In the former case, *E*_GABA_ was fixed at -65 mV, while in the latter case, KCC2 activity was reduced to 33% of its normal level. With weak excitatory and inhibitory input, spiking was higher in the model with static *E*_Cl_ (Fig. 11E). However, as the frequencies of excitatory and inhibitory inputs were increased, all the mechanisms that contribute to a collapse of *E*_Cl_ (examined above) combined to drive *f*_out_ nonlinearly beyond the value predicted by fixed *E*_Cl_ (Fig. 11E). In short, these results show that *E*_Cl_ cannot be approximated by a single, static value when considering a range of stimulus conditions because of the rich dynamics governing *E*_Cl_ under natural conditions. Those dynamics can only be fully understood by accounting for numerous, interdependent biophysical processes.

## Discussion

In this study, we built a neuron model that incorporates multiple processes controlling ion flux in order to investigate how interactions between those processes influence GABA_A_R-mediated inhibition. This was prompted by the recognition that conventional neuron models make oversimplifying assumptions (*e.g.* reversal potentials are temporally invariant and spatially uniform or consider changes in only one ion specie) that are likely to be particularly consequential for GABA_A_R-mediated inhibition. For instance, experiments have shown that *E*_GABA_ can shift during the course of sustained GABA_A_R input [2], [42], that *E*_GABA_ is not uniform across different regions of the same neuron (our results and [26]–[28], [46]) and that *E*_K_ has an important impact on Cl^−^ dynamics. Computational simulations are an ideal tool for investigating questions related to electrodiffusion and interaction between multiple ion species as well as for making predictions to guide subsequent experiments, but the accuracy of those simulations depends on the accuracy of the starting model. With that in mind, we built a neuron model that tracked [Cl^−^] changes as well as other ions that interact with [Cl^−^] homeostasis. Our model accurately reproduced activity-dependent decrease of IPSC amplitude, including differential decrease depending on the site of synaptic input and the compartment geometry [1], [47]. Our model also reproduced spatial variations in *E*_GABA_ and its dependence on the interplay between strength of cotransporter activity and spatial distribution of GABA_A_R input. Having thus validated the model, we explored several other questions.

Upregulation of KCC2 has been linked with the hyperpolarizing shift in *E*_GABA_ observed during early development [7], [20], [45], [48]. Likewise, downregulation of KCC2 has been linked with the depolarizing shift in *E*_GABA_ seen in various disease states [16], [49], [50]. However, the relationship between KCC2 and *E*_GABA_ has not heretofore been quantitatively explored. Simulations in our electrodiffusion model showed that that relationship is highly nonlinear: Reducing KCC2 activity caused a dramatic depolarizing shift in *E*_GABA_, whereas increasing KCC2 activity above normal levels had only a small effect on *E*_GABA_. The reason is that KCC2 already operates near its equilibrium point under normal conditions [51]. These observations suggest that therapies aiming to restore depleted KCC2 levels should not cause excessively strong GABA_A_R-mediated inhibition if KCC2 overshoots its normal level. Moreover, the importance of investigating KCC2 regulation as a therapeutic target is emphasized by the observation that increasing the frequency or duration of GABA_A_R input cannot effectively compensate for disinhibition caused by KCC2 depletion since activity-dependent accumulation of intracellular Cl^−^ is increased under those conditions. In fact, our simulations illustrate how the optimal rate and time course of GABA_A_R input mutually influence each other and also depend on the level of KCC2 activity. Those observations help to explain why drugs that act by increasing GABA_A_R input have variable effects on the treatment of pathological conditions involving disrupted Cl^−^homeostasis, e.g. in neuropathic pain or epilepsy. While administration of benzodiazepines has some efficacy at reversing tactile allodynia in neuropathic pain models, beyond a certain dose, they become counterproductive and enhance allodynia [52], [53]. This bell shaped response to benzodiazepines on neuropathic pain follows directly the predictions from our model (Fig. 8).

Beyond helping understand pathological conditions, our model also provides insight into synaptic inhibition under normal conditions. The importance of interactions between Cl^−^ diffusion and transmembrane Cl^−^ flux became apparent when we considered the temporal dynamics of [Cl^−^]. Simulations revealed that Cl^−^ accumulation near a highly active synapse is rapidly redistributed by intracellular diffusion, whereas Cl^−^ extrusion via KCC2 tends to act more slowly. The large volume of the soma keeps somatic [Cl^−^]_i_ relatively stable, in contrast to dendrites where diffusion is limited by the small diameter of the compartment. Thus, on short time scales, the soma acts as a Cl^−^ sink. It follows that the extent of Cl^−^ accumulation in dendrites does not only depend on the diameter of the dendrite, but also on the distance of the synapse from the soma. Since the dendrite diameter tends to decrease with the distance from the soma, the effects on diffusion are cumulative. As a result, diffusion is responsible for redistributing (and thus mitigating) transient, local changes in Cl^−^ load, while KCC2 level controls the steady-state balance of Cl^−^ influx and efflux. Thus, the faster dynamical collapse of *E*_GABA_ that occurs upon repetitive GABA_A_R input to distal dendrites results from limited diffusion rather than from inefficiency of Cl^−^ extrusion.

xThe functional impact of this result is that distributed synaptic input is more effective than clustered input, especially on distal dendrites where longitudinal Cl^−^ diffusion is particularly restricted. The more labile Cl^−^ gradient in distal dendrites causes a rapid collapse of GABA_A_R-mediated hyperpolarization upon repetitive input, which limits its ability to influence somatic integration especially because, although remote current sources can hyperpolarize the soma, remote conductances do not cause shunting in the soma [1]. This implies that multiple GABAergic connections originating from the same presynaptic cell will be more effective if those synapses are distributed on different dendritic branches. It is interesting to note that this corresponds to the morphological arrangement observed in several systems [54]. This broad distribution contrasts the clustering of axo-axonic synapses that necessarily occurs when a presynaptic cell forms multiple synapses on a postsynaptic neuron's soma and AIS [54], [55]. In the latter case, dynamical collapse of *E*_GABA_ does not occur because the soma acts as a Cl^−^ sink.

The functional impact of the standing [Cl^−^]_i_ gradient along the somato-dendritic axis resulting from the interplay between background GABA_A_R input and cotransporter activity may lead, under certain conditions, to differential impact of distal dendritic vs. somatic GABAergic synaptic input such as, for example, concurrent dendritic GABA_A_-mediated excitation and somatic inhibition [1].

In addition to Cl^−^ dynamics, one must keep in mind that Cl^−^ flux does not occur independently from other ion species. For example, Cl^−^ influx through GABA_A_R is coupled with HCO_3_^−^ efflux. The relationship between Cl^−^ flux and HCO_3_^−^ flux is crucial for explaining how the net current through GABA_A_R can invert as Cl^−^ accumulates intracellularly [2], [44]. Beyond causing a given shift in *E*_GABA_, the HCO_3_^−^ efflux has consequences on the dynamics of the system. Without HCO_3_^−^ efflux, Cl^−^ influx would rapidly stabilize when membrane potential reached *E*_GABA_ because *E*_GABA_ would equal *E*_Cl_. However, due to HCO_3_^−^ efflux, and given that *E*_GABA_ is less negative than *E*_Cl_, intracellular Cl^−^ continues to accumulate when the membrane potential initially reaches *E*_GABA_. In the absence of other extrinsic factors and during sustained GABA_A_R input, intracellular Cl^−^ accumulation and membrane potential drift would progress until *E*_Cl_  =  *E*_GABA_  =  *E*_HCO3_. This progression may, however, be prevented by the influence of other intrinsic currents. In any case, HCO_3_^−^ efflux effectively delays stabilization of the system until a more depolarized membrane potential is reached, which can make a crucial difference for whether or not membrane potential increases above spike threshold (see below). Consistent with these observations, a recent study showed that blocking carbonic anhydrase (and thereby presumably reducing HCO_3_^−^ efflux through GABA_A_R) can mitigate some of the behavioral manifestations of neuropathic pain thought to arise from KCC2 downregulation [52]. Moreover, based on their common reliance on HCO_3_^−^, regulation of [Cl^−^]_i_ competes with regulation of intracellular pH on long time scales (tens of seconds to minutes) consistent with experimental observations [3], [24], [56]. One functional consequence of this is that intracellular Cl^−^ accumulation (and, by extension, possibly the loss of KCC2 expression in pathological conditions) may act as a protective mechanism to prevent an excessive drop in intracellular pH during sustained GABA_A_R input.

The relationship between pH and Cl^−^ homeostasis may also be relevant to recent controversies regarding the necessity of ketone bodies for maintenance of *E*_GABA_ in the developing nervous system [57]–. Given the HCO_3_^−^ dependence of the beta-hydroxybutyrate effect on *E*_GABA_ in these experiments, it has been proposed that the explanation may reside in the fact that beta-hydroxybutyrate, lactate or pyruvate act as weak organic acids, thus acidifying the neuronal cytoplasm and reversing Cl^−^/HCO_3_^−^ exchange; this counteracts the drop in [HCO_3_^−^]_i_ due to acidification but, by the same token, it lowers [Cl^−^]_i_ and drives *E*_GABA_ to a more negative value [59], [61]. Our simulations are consistent with this explanation.

Given the coupled efflux of Cl^−^ and K^+^ through KCC2, Cl^−^ extrusion happens at the expense of extracellular K^+^ accumulation. This may appear counter-productive as extracellular K^+^ accumulation counteracts inhibition and plays a role in the onset of epilepsy [62], [63]. However, we found that under physiological conditions, K^+^ efflux through KCC2 is offset by the fact that KCC2 activity enhances inhibition, thus decreasing firing rate and reducing K^+^ efflux via transmembrane channels. The net effect is a reduction of excitability because K^+^ efflux via transmembrane channels is larger than via KCC2. We found that this negative feedback stabilizes [K^+^]_o_ over a wide range of KCC2 activity. Disrupting this homeostasis requires sustained input from extrinsic factors. For example, intense GABAergic activity, which can maintain a continuous Cl^−^ load leading to a large and sustained K^+^ efflux through KCC2, has been observed during giant depolarizing potentials [46]. Likewise, excessive spiking yields continuous extracellular K^+^ accumulation, which renders KCC2 inefficient, causing a collapse of inhibition due to intracellular Cl^−^ accumulation.

Another interesting observation was that membrane depolarization tends to encourage intracellular Cl^−^ accumulation because Cl^−^ influx through GABA_A_R depends on Cl^−^ driving force, which is increased by depolarization. The consequences are profound: If sustained GABA_A_R input fails to prevent depolarization caused by concurrent excitatory input, the resulting depolarization will accelerate Cl^−^ influx, which in turn further reduces the GABA_A_R-mediated outward current, thus supporting a positive-feedback cycle of failing inhibition. If the membrane potential reaches the spike threshold under these conditions, spike generation compounds the positive feedback process leading to an absolute failure of inhibition having potentially catastrophic consequences with respect to the neuron's response to stimulation. The only way for a neuron to avoid entering this vicious cycle is to regulate [Cl^−^]_i_, through Cl^−^ extrusion via KCC2.

In summary, we built a neuron model that incorporates multiple processes controlling the flux of different ion species in order to investigate how interactions between those processes influence inhibition mediated by GABA_A_R. Many of those processes cooperate or compete with one another, thus producing nonlinearities. The most dramatic of those is arguably the catastrophic failure of inhibition that can develop when depolarization and spiking conspire with Cl^−^ accumulation to form a positive feedback loop. As demonstrated in this study, such details may be critical for understanding important aspects of synaptic inhibition, in particular, for understanding why and how inhibition fails under certain pathological conditions.

## Methods

We built a conductance-based model of a whole neuron using the NEURON simulation environment [64] (model code will be made available at ModelDB). The model is composed of 30 dendritic compartments unless otherwise indicated, one somatic compartment, one compartment for the axon initial segment (AIS), and 10 myelinated axonal compartments separated by nodes of Ranvier. Details of the geometry are summarized in Figure 1A. Ionic currents flowing through channels, pumps and cotransporters were computed at each time step in order to update the membrane potential according to 
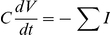
where C is membrane capacitance of the neuron compartment and the sum is taken over synaptic currents, current through voltage gated channels and electrogenic Na-K ATPase. Transmembrane ion flux due to those currents was also calculated. Moreover, longitudinal and radial diffusion were incorporated into the model in order to account for intracellular ion gradients. Likewise, extracellular ionic diffusion was taken into account as well as chemical reactions that produce the various ion types (see below). Transmembrane ion flux, ion movement through diffusion, and ion generation through chemical reactions (Fig. 1B) were all taken into account when updating the concentration of ion specie *x* in each compartment at each time step according to the differential equation 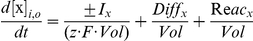, where *F* is the Faraday constant, *z* is the ion valence, *Vol* is the compartment volume, *Reac_x_* is a term accounting for chemical reactions involving ion species *x* and *Diff_x_* is a term modeling the *electrodiffusion* of ion *x* [65], [66]. Synaptic events are expressed in currents, but the membrane potential was not clamped, consistent with realistic conditions. This is of importance since invasive cell manipulations have been shown to alter the nature (inhibitory or excitatory) of GABA_A_ mediated input [67].

### Channels

Ion currents obey the equation 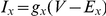 where *E_x_* denotes the reversal potential for ion *x* and *g_x_* is the channel conductance with respect to ion *x*. Reversal potentials were continuously updated during the simulation using the Nernst equation 
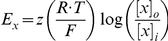
 where *R* is the perfect gas constant and *T* is absolute temperature, which was taken to be 310°K (37°C). Because GABA_A_ receptors pass both Cl^−^ and HCO_3_^−^ anions in a 4∶1 ratio [24], *E*_GABA_ was calculated using the Goldman-Hodgkin-Katz equation 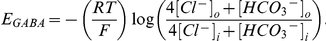



Each of these ionic currents was taken into account for computing change in concentration of their respective ion species and their sum yielded the net current used to update the membrane potential.

Synaptic input was modeled as a Poisson process. Each inhibitory synapse was activated at a mean frequency of 0–10 Hz and each excitatory synapse was activated at a mean frequency of 0–2 Hz. Unless otherwise stated, the maximal conductance of inhibitory synapses was 1±0.3 nS (mean ± standard deviation) and kinetics were modeled as instantaneous rise and exponential decay with τ_IPSC_ of 30 ms [30], [68]–[70]. GABA_A_R synaptic density was 60 synapses per 100 µm^2^ in the AIS, 40 synapses per 100 µm^2^ in the soma and 12 synapses per 100 µm^2^ in the dendrites. Density of excitatory synapses was 60 synapses per 100 µm^2^ in dendrites and no excitatory synapses were present elsewhere [21], [30]. Unless otherwise stated, the maximal conductance of excitatory synapses was taken to be 0.5±0.2 nS (mean ± standard deviation) and the kinetics were modeled as an instantaneous rise and exponential decay with τ_EPSC_ of 10 ms.

Hodgkin-Huxley (HH) channels were modeled using parameter values reported by [12]. The voltage-dependant Na^+^ current was given by
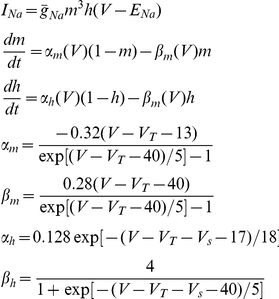



Where *V_T_* = −58 mV and *V_S_* = −10 mV. The voltage gated K^+^ channels were described by
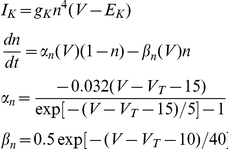



The density of HH channels was 12 mS/cm^2^ in the AIS and 1.2 mS/cm^2^ in soma and dendrites [21], [30]. The model also included K^+^ and Na^+^ leak channels with respective densities of 0.02 mS/cm^2^ and 0.004 mS/cm^2^ in soma, 0.03 mS/cm^2^ and 0.006 mS/cm^2^ in proximal dendrites, 0.1 mS/cm^2^ and 0.02 mS/cm^2^ in distal dendrites, 0.02 µS/cm^2^ and 0.004 µS/cm^2^ in axon internodes, and 15 mS/cm^2^ and 3 mS/cm^2^ in axon nodes as described in [21], [30].

For some simulations, we added other types of conductances to account for the many possible types of spike generating mechanisms. Namely, we added non-inactivating Ca^2+^-activated K^+^ channels and persistent Na^+^ channels to test spike reducing and spike enhancing mechanisms, respectively. The Ca^2+^-activated K^+^ channels obey the following sets of equations
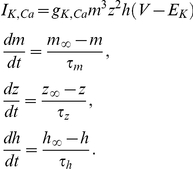



Where the auxiliary functions are defined by
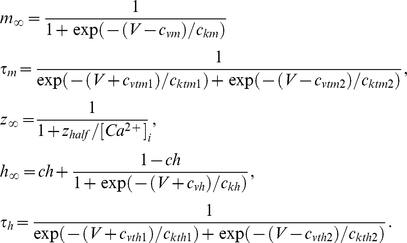



With the constants defined as c_vm_ = 28.9 mV, c_km_ = 6.2 mV, ctm = 0.000505 s, c_vtm1_ = 86.4 mV, c_ktm1_ = -10.1 mV, c_vtm2_ = -33.3 mV, c_ktm2_ = 10 mV. τ_z_ = 1 s, ch = 0.085, c_vh_ = 32 mV, c_kh_ = -5.8 mV, c_th_ = 0.0019 s, c_vth1_ = 48.5 mV, c_kth1_ = -54.2 mV, c_vth2_ = -54.2 mV, c_kth2_ = 12.9 mV.

The persistent Na^+^ channels were described by the following set of equations:
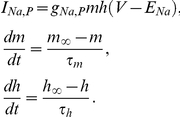



Where the auxiliary functions are defined by
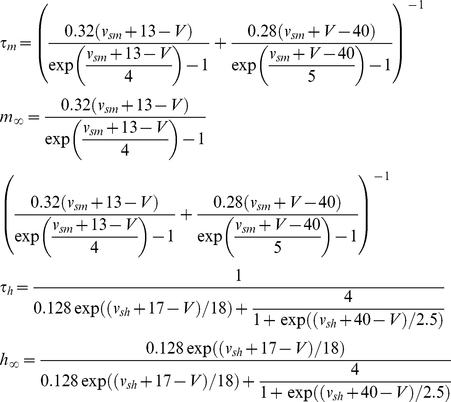



Where the constants are given by v_sm_ = −2 mV and v_sh_ = −5 mV.

Finally, to account for non-synaptic, tonically activated Cl^−^ conductance, in some simulations we added GABA_A_ leak channels with the same ratio of Cl^−^:HCO_3_^−^ permittivity as the synaptic channels, the density of such channels was 0.003 mS/cm^2^ in soma, 0.0045 mS/cm^2^ in proximal dendrites, 0.015 mS/cm^2^, 0.003 µS/cm2 in axon internodes and 2.3 mS/cm^2^ in axon nodes.

### Sodium-potassium pump and cation-chloride cotransporters

The Na^+^-K^+^-ATPase pump uses the energy from hydrolysis of one ATP molecule to pump three Na^+^ ions out of the neuron and two K^+^ ions inside. The activity of this pump is dependent on [K^+^]_o_ and [Na^+^]_i_ as well as on the membrane potential as observed in [71]. The Na^+^ current through the pump is given by the following equations
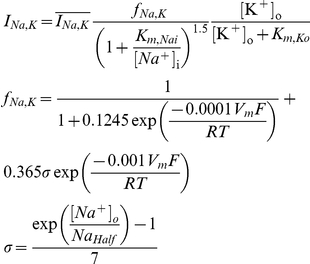

[72], [73]. With K_m,Ko_ = 1.5 mM, K_m,Nai_ = 10 mM and Na_Half_ = 20 mM. The outgoing Cl^−^ flux though KCC2 was modeled according to [2], [4] by 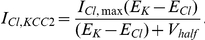



The K^+^ current through KCC2 was assumed to be equal in absolute value but opposite in polarity to the Cl^−^ current so that net current through KCC2 was equal to zero. The maximal Cl^−^ current going through KCC2 was taken to be *I_Cl,max_* = 0.3 mA/cm^2^ for the normal activity level. This value was chosen to give *E*_Cl_  =  −80 mV when mean synaptic input frequencies were 3.2 Hz and 1.6 Hz for inhibition and excitation, respectively. This value also turned out to yield maximal Cl^−^ clearance rates near 10 mM/s, consistent with experimental data [4]. The value of the driving force (*E*_Cl_-*E*_K_) at which the Cl^−^ current through KCC2 reaches its half maximal value (*V*_half_) was set to 40 mV. This corresponds to [Cl^−^]_i_  =  15 mM under the assumption that [Cl^−^]_o_  =  120 mM and *E*_K_  =  -95 mV.

In some simulations, we also modeled NKCC1 activity in the AIS. The Cl^−^ influx through NKCC1 was modeled by
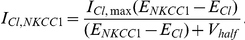



Where *E*_NKCC1_ is the value of *E*_Cl_ at which Cl^−^ flow through NKCC1 reverses direction and is given by *E*_NKCC1_  =  (*E*_K_+*E*_Na_)/2. The maximal Cl^−^ current going through NKCC1 was taken to be *I_Cl,max_*  =  0.3 mA/cm^2^ for the normal activity level which was taken to equal the value obtained for maximal current through KCC2. Na^+^ and K^+^ currents through NKCC1 were each half of *I_Cl,NKCC1_* so that net current through NKCC1 was equal to zero.

Finally, in some simulations we also modeled the Cl^−^/HCO_3_^−^ exchanger which was assumed to be ubiquitous in the neuron and uniformly distributed on the membrane. Kinetics of the exchanger were described by the following simplified equation.
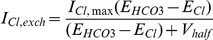
where *ICl,max* =  0.1 mA/cm^2^ and *Vhalf* was set to 50 mV. The HCO3^-^ and Cl^-^ currents through the exchanger were taken to be equal in amplitude but opposite in direction so that the exchange process is electroneutral.

### Bicarbonate and pH management

Since HCO_3_^−^ ions also flow through GABA_A_R channels (see above), it was important to model the ionic fluxes and reactions regulating [HCO_3_^−^]. Intracellular HCO_3_^−^ loss due to outgoing flux via GABA_A_R is compensated by the carbonic anhydrase-catalyzed reaction 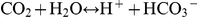 [3], [44], [56], [74]. Since the diffusion of CO_2_ gas through the membrane is faster than ionic fluxes through channels, we treated pCO_2_ as constant. The equilibrium constant of the reaction was 10^−6.35^ M and the rate constant for CO_2_ hydration was 10^6^ sec^−1^ [75]. Since the above reaction produces a drop in pH [42], [44], [56] by causing intracellular H^+^ accumulation, we also modeled the reaction between H^+^ and the main buffering ion H_2_PO_4_^−^ such that H^+^ buffering occurred through the reaction 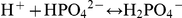. Although other reactions play important roles in pH buffering, we kept the model as simple as possible while preserving the global value of pH buffering capacity, estimated to be 25–30 mMol/pHU [76], [77]. Buffering reactions are responsible for maintaining the pH value constant at short time scales (∼100 ms), but proton extrusion via exchangers plays an important role on longer time scales (>10s). For the sake of simplicity, we limited ourselves to modeling the Na^+^-H^+^ exchanger such that the proton flux was given by
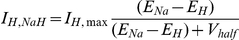
which is a generic scheme for transporters. We used *I_H,max_*  =  0.03 mA/cm^2^ and V_half_  =  10 mV. These values were chosen to make the model consistent with the global proton extrusion rate in healthy neurons that has been measured to be 0.04 pHU/s [78]–[80].

### Electrodiffusion

An important and novel feature of the model is that the intra- and extracellular concentrations of K^+^, Na^+^, Ca^2+^, Cl^−^, HCO_3_^−^ as well as intracellular concentrations of H^+^, HPO_4_^2−^ and H_2_PO_4_^−^ were treated as dynamical variables updated in each compartment at each time step. Each compartment was divided in four concentric annulus-shaped subcompartments to account for radial diffusion. Diffusion coefficients were assumed to be the same as in water (in 10^−5^ cm^2^/s): 2.03 for Cl^−^, 1.33 for Na^+^, 1.96 for K^+^, and 9.33 for H^+^ [81]. Longitudinal *electrodiffusion* is described by the equation 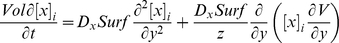 where *y* stands for the longitudinal axis, *D*_x_ for the diffusion coefficient with respect to ion specie *x*, *Vol* for section volume and *Surf* for the surface of the cross section [66]. The first term is due to pure diffusion while the second term accounts for the electrical force acting on the ions. The second term was used only to compute electrodiffusion between outer annuli of dendritic sections and was set to 0 for inner annuli, consistent with the fact that electrical field extends only to a thin region near the membrane. This is because the membrane act as a capacitor and electric field is known to decrease rapidly [82]. Radial diffusion was computed in a similar way but with *y* representing the radial axis.

Extracellular space was represented as a thin shell (i.e. Frankenhaeuser-Hodgkin or FH space) with equivalent volume equal to one fourth the intracellular volume of the corresponding cell compartment. The inner surface of the FH space communicated with the adjacent intracellular compartment while the outer surface was linked to an infinite reservoir where ion concentrations were assumed to be constant. This modeling takes into account changes in [K^+^]_o_ due only to our cell, and thus does not address changes in [K^+^]_o_ due to network activity. The study of such network related effects is beyond the scope of the current study. The equation used to update extracellular ion concentration is given by 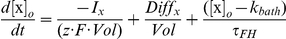 where *z* is the ion valence, *k_bath_* is the concentration of ion *x* in the infinite reservoir and τ_FH_ is the time constant taken to be 100 ms [79], [83].

### Numerical methods

The differential equations were integrated using a forward Euler method with a time step of 0.05 ms. Several preliminary simulations showed this time step to be both sufficiently small for accurate equation solving, while sufficiently large for reasonably fast computing. Initial intracellular concentrations were (in mM): [Cl^−^]_i_ = 6, [K^+^]_i_ = 140, [Na^+^]_i_ = 10, [HCO_3_^−^]_i_ = 15 [H_2_PO_4_^−^]_i_ = 30 and [HPO_4_^2−^]_i_ = 30. Initial extracellular concentrations were in (mM) [Cl^−^]_o_ = 120, [K^+^]_o_ = 3, [Na^+^]_o_ = 45 and [HCO_3_^−^]_o_ = 25 [81]. Preliminary simulations were conducted to determine initial concentrations such that they were stable under normal conditions (in the absence of high frequency synaptic input). For simulations in which the value of maximal Cl^−^ current through KCC2 (*I_Cl,KCC2_*) was different than the normal one stated above (*I_Cl,max_* = 0.3 mA/cm^2^), different initial values of [Cl^−^]_i_ were used in order to start the simulation near steady state, as determined by preliminary testing. Unless stated otherwise, simulations were run for 200 s of simulated time, short enough to allow manageable simulations and long enough to allow collection of sufficiently large data sample to insure relevance of mean values.

### Cell cultures

Dissociated hippocampal neurons from Sprague-Dawley rats were prepared as previously described [84] plated at P0 to P2 at a density of approximately 500–600 cells/mm^2^ and imaged after 21–30 days in vitro (DIV). Glial proliferation was stopped at 5 DIV with Ara-C.

### Chloride imaging

Cells were loaded in a 5 mM solution of the Cl^−^ indicator MQAE (*N*-6-methoxyquinolinium acetoethylester; Molecular Probes) for 30 min at 37 °C [85]. Prior to observation, cells were transferred to a perfusion chamber and bathed in bicarbonate-buffered saline containing: 100 NaCl, 2.5 KCl, 1 NaH_2_PO_4_, 26 NaHCO_3_, 1 MgCl_2_ and 1.2 CaCl_2_. Muscimol (100 µM, Tocris), furosemide (50-200 µM, Sigma), kainic acid (50 nM, Tocris), VU 0240551 (25-50 µM, Tocris) and bicuculine (100 µM, Sigma) were selectively added as described in the result section. Upon addition of drugs, cells were allowed to adjust for 10–20 minutes before a steady-state image of their Cl^−^ contents was taken.

Fluorescence lifetime images of MQAE were acquired using a Becker & Hickl SPC-830 module coupled to a Zeiss LSM 510 microscope. MQAE was excited using a femtosecond pulsed Ti-Sapphire laser tuned at 760 nm (Chameleon Ultra, Coherent), through a 40X water-immersion objective (Zeiss, 0.8 NA). Fluorescence lifetime data was collected through the non-descanned port of the microscope using a band-pass filter (469/35 nm, Semrock) coupled to a laser block (short-pass 750 nm; Semrock). Photon emission was detected using a PMC-100-1 photosensor (Hamamatsu). Lifetime in each cell compartment was calculated and extracted using SPCImage software (Becker & Hickl). Lifetime in the cell body was averaged over the total cell body area excluding the nucleus region, whereas in the dendrites it was averaged in segments of 4 µm over 120 µm of dendrite length. Fluorescence lifetime measurements were used because they are not sensitive to dye concentration (peak intensity) in the range we are using [85], [86]. The lifetime measurements are thus not affected by differences in dye loading from cell to cell or by volume changes that could occur in different cell compartments (Fig. 2A). The Cl^−^ dependence of MQAE lifetime is described by the Stern-Volmer relation (τ_0_/τ  =  1 + Ksv [Cl^−^]_i_), where τ_0_ is the fluorescence lifetime in 0 mM Cl^−^, and Ksv, the Stern-Volmer constant, is a measure of the Cl^−^ sensitivity of MQAE (Fig. 2B).

### Chloride calibration in cells

For calibration of absolute Cl^−^ concentrations, the fluorescence lifetime of MQAE-loaded cells was measured in the presence of different known extracellular [Cl^−^] (8, 15 or 20 mM) in the bath. To dissipate the Cl^−^ gradient across the membranes, 20 µM tributyltin (Cl^−^-OH exchanger) was used and 20 µM nigericin (K^+^-H^+^ exchanger) was added to clamp the intracellular pH using high K^+^ driving force while Cl^−^ changes. Calibration solutions contained (in mM) KCl and KNO_3_ (140 K^+^ total with desired amount Cl^−^), 10 D-glucose, 10 HEPES, 1.2 CaCl_2_, 1 MgCl_2_, pH adjusted to 7.2 using KOH.

### Immunocytochemistry

Primary hippocampal cultures (5 and 28 DIV) were fixed for 10 min with 4% paraformaldehyde and then permeabilized for 45 min with 0.2% triton in 10% normal goat serum (NGS). Primary antibody incubations were performed overnight at 4 °C using a polyclonal marker of KCC2 (Rabbit anti-KCC2 1∶500, Upstate) in the presence of 5% NGS. Alexa 546 conjugated secondary antibodies (1∶750; Invitrogen, Eugene, OR) were applied for 2 hrs at room temperature. Images were obtained on the Zeiss LSM 510 microscope using a 63X/1.4NA oil objective (Zeiss).

### Spatial intensity distribution analysis (SpIDA)

SpIDA is a recently developed analysis method that can resolve concentration of mixtures of different monomeric and oligomeric labels in single fluorescence images by fitting its intensity histogram. Precise details of the technique and detector calibration are presented in [33]. Briefly, the intensity histogram fitting function for a system with density *of* N particles is:
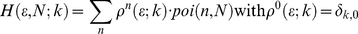



Where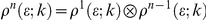 with 
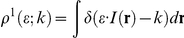
_._I(**r**) is the illumination intensity profile of the excitation laser, ε represents the quantal brightness of a single fluorescent particle, and *k* is the probability of observing an *intensity* of light (assumed to be proportional the number of photons emitted). *H* is normalized over all intensity values so the integral over *k* gives one. A constant factor, *A*, is introduced, which is the number of pixels in an analyzed region of the image where the fluorescent particles are distributed. This allows for the fit of an image intensity histogram to be performed. Three parameters are fit: the number of pixels (*A*), the fluorescent particle density (*N* particles per laser beam effective focal volume) and the quantal brightness (ε intensity units, iu, per unit of pixel integration time). In confocal laser scanning microscopes, the fluorescence intensity is measured using photon multiplier tubes (PMTs), and the number of collected photoelectrons is a function of the polarization voltage.

If dimers are present in the sample, they will yield quantal brightness of 2ε. When the monomer and dimer populations are mixed within the same region in space, the total histogram becomes the convolution of the two individual distributions: 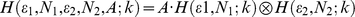



To obtain accurate results, noise characteristics of the detector also has to be studied and taken into account in the analysis, See [33] for complete analysis.

For each sample, an optimal setting of the laser power and PMT voltage was chosen to minimize pixel saturation and photobleaching. The CLSM settings were kept constant for all samples and controls (Laser power, filters, dichroic mirrors, polarization voltage, scan speed). Acquisition parameters were always set within the linear range of the detector which was determined by calibration [33]. All the images were 1024×1024 pixels with pixel size of 0.115 µm and 9.1 µs pixel dwell time. The z-stacks were taken by optical sectioning with a *z* step of 0.5 µm per image.

### Ethics statement

All experiments were performed in accordance with regulations of the Canadian Council on Animal Care.
